# Functional Hyperconnectivity and Task-Based Activity Changes Associated With Neuropathic Pain After Spinal Cord Injury: A Pilot Study

**DOI:** 10.3389/fneur.2021.613630

**Published:** 2021-06-10

**Authors:** Shana R. Black, Jace B. King, Mark A. Mahan, Jeffrey Anderson, Christopher R. Butson

**Affiliations:** ^1^Biomedical Engineering, University of Utah, Salt Lake City, UT, United States; ^2^Scientific Computing and Imaging Institute, University of Utah, Salt Lake City, UT, United States; ^3^Radiology and Imaging Sciences, University of Utah, Salt Lake City, UT, United States; ^4^Neurosurgery, University of Utah, Salt Lake City, UT, United States; ^5^Neurology, University of Utah, Salt Lake City, UT, United States; ^6^Psychiatry, University of Utah, Salt Lake City, UT, United States

**Keywords:** functional connectivity, resting-state fMRI, task-based fMRI, functional magnetic resonance imaging, fMRI, spinal cord injury, neuropathic pain

## Abstract

Neuropathic pain (NP) is a devastating chronic pain condition affecting roughly 80% of the spinal cord injury (SCI) patient population. Current treatment options are largely ineffective and neurophysiological mechanisms of NP are not well-understood. Recent studies in neuroimaging have suggested that NP patients have differential patterns of functional activity that are dependent upon the neurological condition causing NP. We conducted an exploratory pilot study to examine functional activation and connectivity in SCI patients with chronic NP compared to SCI patients without NP. We developed a novel somatosensory attention task to identify short term fluctuations in neural activity related to NP vs. non-painful somatosensation using functional magnetic resonance imaging (fMRI). We also collected high-resolution resting state fMRI to identify connectivity-based correlations over time between the two groups. We observed increased activation during focus on NP in brain regions associated with somatosensory integration and representational knowledge in pain subjects when compared with controls. Similarly, NP subjects showed increased connectivity at rest in many of the same areas of the brain, with positive correlations between somatomotor networks, the dorsal attention network, and regions associated with pain and specific areas of painful and non-painful sensation within our cohort. Although this pilot analysis did not identify statistically significant differences between groups after correction for multiple comparisons, the observed correlations between NP and functional activation and connectivity align with *a priori* hypotheses regarding pain, and provide a well-controlled preliminary basis for future research in this severely understudied patient population. Altogether, this study presents a novel task, identifies regions of increased task-based activation associated with NP after SCI in the insula, prefrontal, and medial inferior parietal cortices, and identifies similar regions of increased functional connectivity associated with NP after SCI in sensorimotor, cingulate, prefrontal, and inferior medial parietal cortices. This, along with our complementary results from a structurally based analysis, provide multi-modal evidence for regions of the brain specific to the SCI cohort as novel areas for further study and potential therapeutic targeting to improve outcomes for NP patients.

## Introduction

Neuropathic pain (NP) is a spontaneous, chronic pain condition caused by a lesion or disease of the nervous system (1). It is reported to affect ~80% (2) of the estimated 294,000 people currently living with spinal cord injury (SCI) and the roughly 18,000 people per year who sustain a SCI in the United States alone (3). NP remains complex and difficult to treat for the SCI patient population, due in part to other secondary conditions of SCI and a lack of a physiological mechanistic understanding of NP in general (4). Functional neuroimaging provides an opportunity to better understand how the brain actively and passively processes NP. However, a majority of past NP studies have used healthy, ambulatory control populations. SCI itself is known to result in functional and structural reorganization within the central nervous system (5–10), limiting our ability to draw strong conclusions from these studies with healthy controls. Although there are a growing number of studies that do compare those who develop NP after SCI to those who do not, a clear understanding of neurological activity related to NP in the SCI population has yet to be developed.

Previous neuroimaging studies have used resting state functional magnetic resonance imaging (rs-fMRI) to identify activity-based differences between those with and without chronic pain in other patient populations. Many of these studies have shown a decrease in default mode network activity, increased correlation between typically anticorrelated networks, and increased functional activity in somatomotor, insular, frontal, and medial parietal regions in patients with various pain conditions compared to controls (11–15). It has also been shown that such neurological signatures seem to be specific to the cause of NP (16), with distinct differences in resting state activity between populations with different primary diagnoses, such as diabetic neuropathy and failed back surgery syndrome (15–18). Task-based fMRI (t-fMRI) has also been used in an attempt to elucidate neurological correlates of pain perception in chronic pain populations (19–21). However, these studies have utilized elicited pain paradigms, which are likely more applicable to the acute pain response rather than identifying activation patterns related to the chronic pain state. Further, those with SCI have been shown to have altered pain modulation properties in response to external stimuli and the relationship of these neurological changes to NP perception is unknown (22).

In this pilot study, we use t-fMRI and rs-fMRI to identify activation and connectivity differences in SCI subjects with chronic NP compared to SCI controls without NP. We have developed a novel task in an attempt to identify short-term activation differences between painful and non-painful somatosensation without external stimulation. We also sought to identify functional connectivity differences in those with NP using rs-fMRI to identify activity-based correlations between cortical and subcortical brain regions over time. In addition to this fMRI analysis, we have completed a complementary study of structural connectivity and gray matter volume changes associated with NP after SCI in the same cohort^1^. These studies together provide multi-modal evidence for brain regions specifically correlated with NP after SCI that could be used to guide future development of targeted neurological intervention options to improve pain outcomes for these patients.

## Materials and Methods

### Subject Selection and Collection of Medical History and Demographics

Subjects were selected via medical record review based on the following inclusion and exclusion criteria: (1) individuals must have sustained a traumatic SCI at least 1 year prior to enrollment in the study; (2) individuals must be between 18 and 45 years of age at the time of enrollment; (3) medical records must show that individuals have an American Spinal Cord Injury Association Impairment Scale (AIS) classification of either A or B, indicating that they do not have motor function below their level of SCI; (4) individuals cannot have any medical history of diabetes, cancer, amputation, brain injury, stroke, or any neurological injury or condition other than SCI; (5) individuals do not report any pain condition, such as arthritis, other than NP; (6) individuals do not have any conditions with which an MRI would be unsafe. All subjects provided informed consent to undergo study procedures and the full study protocol was approved of by the University of Utah Institutional Review Board.

In addition to a review of medical records for each participant, each subject completed a questionnaire that confirmed SCI date, level, and method of injury, as well as current medical diagnoses, medical history, and current medications. Subjects also completed the Hospital Anxiety and Depression Scale (HADS) (23). To assess presence and severity of NP, subjects completed three additional pain rating scales. The first of these included a subjective pain description and a numeric pain rating scale (NPRS), for which subjects rated their pain (0–10) at the time of their MRI scan as well as their worst and best pain rating for the 24 h prior to the scan. The second pain rating scale was the Neuropathic Pain Symptom Inventory (NPSI) (24), which asks about various neuropathic symptoms over the previous 24 h, each classified into a NP sub-category and rated on a scale of 0–10. Finally, subjects completed the Brief Pain Inventory (BPI) (25), which captures subject demographic information, pain location and treatment strategies, and quantifies pain severity and functional interference. Subjects with a maximum NPSI sub-score of at least 2 were put in the pain group and those lacking neuropathic symptoms were placed in the control group.

### Image Acquisition and Analysis

All imaging for this study was obtained using a 64-channel head coil on the same Siemens PRISMA 3T system in the Utah Center for Advanced Imaging Research (UCAIR) at the University of Utah. Prior to any analysis, subjects' structural volumes, obtained using an MP2RAGE imaging sequence with 1 × 1 × 1 mm voxel resolution, 5,000 ms TR, and 2.93 ms TE, were realigned to correct for motion between slices, segmented, and registered to MNI space. Pre-processing and analysis of structural and functional imaging data was completed using Statistical Parametric Mapping (SPM12) (26) and MATLAB software unless otherwise specified.

#### Task fMRI

**Figure 1:**
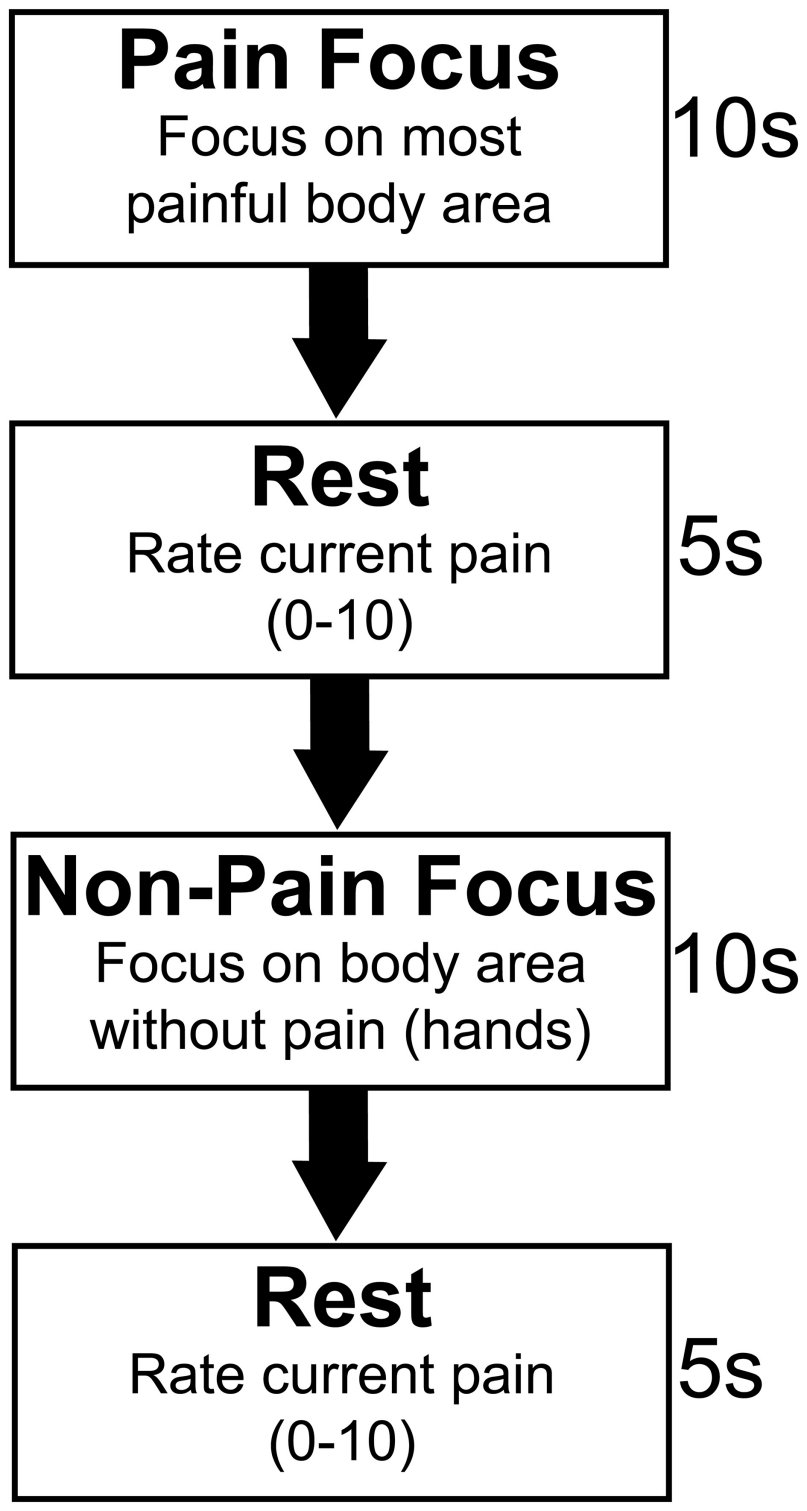
Somatosensory focus task paradigm. Pain subjects were asked to alternate between two active focus periods of 10 s. For the first focus period, the pain focus, subjects were asked to focus only on their most painful area. Subjects were asked to focus on their intrinsic somatosensation in a body area in which they did not have pain symptoms for the second, non-pain focus period. This was the hands for all subjects. Five second rest periods separated focus periods during which time pain subjects verbally rated their pain on a 0–10 rating scale. Control subjects were asked to focus on the intrinsic somatosensation in the same two body areas as their age, sex, and injury level matched pain group counterpart.

Following acquisition of structural imaging, subjects underwent multi-band echoplanar t-fMRI which consisted of a somatosensory focus task lasting 8 min and 26.3 s, resulting in 670 image volumes with 2 × 2 × 2 mm voxel resolution, 740 ms TR, 33.2 ms TE, a multi-band acceleration factor of eight, and a flip angle of 52 degrees. The task, outlined in Figure 1, consisted of 10 s focus periods separated by 5 s rest periods. During the focus periods, pain group subjects were asked to alternate between focusing on the body area in which their pain was highest (pain focus state), and their hands, a body area in which none of the subjects had pain symptoms (non-pain focus state). Pain group subjects were also asked to verbally rate their pain from 0 to 10 between each focus period. Verbal ratings were obtained, rather than using a finger-controlled mechanism, due to the fact that approximately one third of the subjects for this study did not have sufficient motor function to operate a hand held device. Control group subjects were age (±4 years), sex, and injury level (±2 levels) matched to pain group subjects (see Table 1). All three matching criteria were considered when pairing subjects between groups; sex could not vary, level of injury could not vary more than two spinal levels, and age could not vary more than 4 years in either direction between matches. If a subject in one group did not have a corresponding match in the other group, they were excluded from the groupwise comparisons for this analysis. Control group subjects were asked to alternate focusing on the same two body areas as their pain group counterpart. Pain subjects' most painful body region was obtained prior to their MRI and all cueing was presented electronically using E-Prime 3.0 Software (Psychology Software Tools, Pittsburgh, PA).

**Table 1:** Groupwise summary of demographics, SCI statistics, and anxiety, depression, and pain metrics.

**Variable**	**Pain group**	**Control group**
Sex (M/F)	14/6	14/2
Mean age (SD)	21.05 (9.01)	28.75 (6.01)
Tetraplegic	11	11
Paraplegic	9	5
Mean time since SCI in months (SD)	95.25 (86.25)	89 (62.09)
**Method of injury**		
Vehicular	11	10
Fall	2	1
Sports	7	4
Other	0	1
Mean HADS anxiety score (SD)^**^	6.65 (3.36)	2.18 (1.80)
Mean HADS depression score (SD)^*^	5.05 (5.05)	1.94 (1.70)
Mean NPSI maximum subscore (SD)	5.66 (1.94)	0
Mean NPRS at scan time (SD)	3.45 (1.65)	0
Mean BPI severity score (SD)	3.93 (1.83)	0
Mean BPI interference score (SD)	3.59 (2.85)	0

* *Significant difference between pain and control groups (p < 0.005)*.

** *Significant difference between pain and control groups (p < 0.00005)*.

Each subject's t-fMRI volumes were realigned to correct for movement between measurements, high-pass filtered at 128 s, coregistered to that subject's T1 structural imaging, smoothed using a 6 × 6 × 6 mm full width half maximum Gaussian kernel, and registered to MNI space for between subject and group comparisons. We compared blood oxygen level dependent (BOLD) activation differences between pain and control group subjects using an independent two sample *t*-test for the contrast between each focus state (i.e., BOLD activation during pain focus state minus activation during non-pain focus state, indicating increased activation during the pain focus state, was compared between groups), using age, sex, and time since SCI as nuisance covariates and a cluster defining threshold of *p* < 0.05. Only subjects for whom there was a matched control were used for this comparison. We also compared activation differences between the pain and non-pain focus state within the pain group alone using the same statistical methods. Finally, we completed a *post-hoc* analysis to (1) assess any significant activation during either the pain or the non-pain focus state for each group individually and (2) identify differences in the between groups comparison with a non-parametric permutation testing approach using the Statistical non-Parametric Mapping (SnPM) toolbox in SPM12 (27). For the SnPM statistical approach, we repeated the analysis using cluster defining thresholds of *p* < 0.001 and *p* < 0.01.

#### Resting-State fMRI

Two 15-min acquisitions of multiband echoplanar rs-fMRI were obtained for each subject. Each acquisition resulted in 1,220 image volumes with 2 × 2 × 2 mm voxel resolution, 740 ms TR, 33.2 ms TE, multi-band acceleration factor of 8, and a flip angle of 52 degrees. To facilitate motion and artifact correction, the two scans were acquired with opposite phase encoding directions (L-R and R-L). Similar to the t-fMRI time series, volumes were realigned to correct for motion, coregistered to the structural imaging, and registered to MNI space. We also performed voxelwise nuisance regression of motion parameters as well as pulse and respiratory waveforms in the BOLD signal using phase shifted soft tissue correction techniques (28). Specifically, we regressed out the following signal components, each of which was bandpass filtered between 0.001 and 0.1 Hz (28): [1] White matter time series were obtained from the mean time series of voxels within two regions of interest in the bilateral centrum semiovale; [2] CSF time series were obtained from degraded image of the lateral ventricles, removing all voxels not completely surrounded by CSF; [3] Soft tissue time series was obtained from a restriction mask of the extracranial facial soft tissues; [4] Respiration volume per time convolved with respiration response function (29); [5] Respiratory belt measurements integrated over each TR to obtain average position of the chest during each imaging volume; [6] Pulse oximeter, integrated over each TR; [7] Time series obtained from 6 affine realignment parameters from the motion correction step. We further corrected for motion by censoring the time series, removing volumes before and after any head movement >0.2 mm. The time series used for subsequent analysis were comprised of the remaining concatenated volumes for each of the two acquisitions for each subject. We compared head motion between groups using a two-sample *t*-test with the mean head motion for each subject across both rs-fMRI scans.

**Figure 2:**
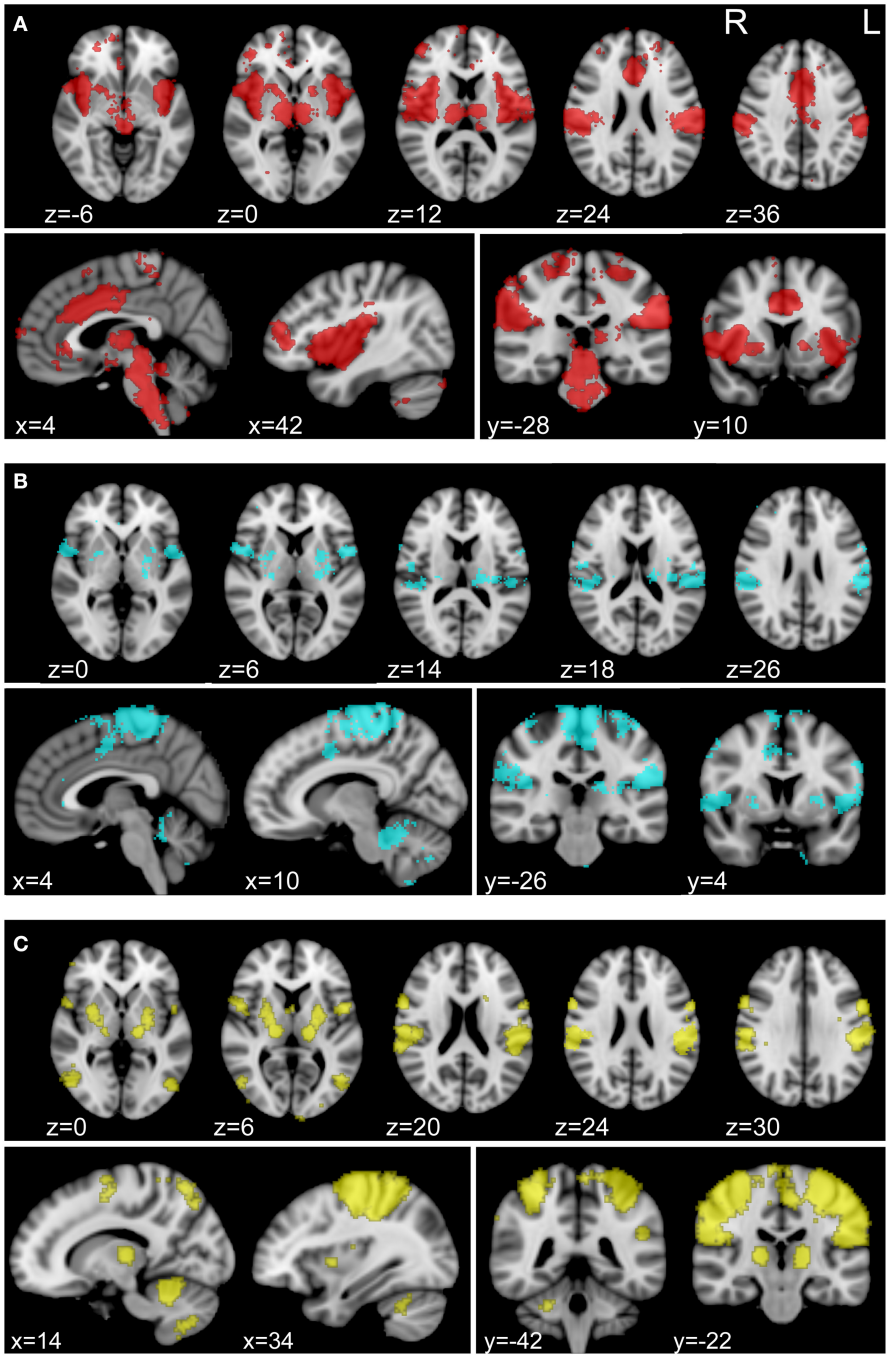
Individually defined association maps used as ROIs for connectivity analysis. Association maps isolated with Neurosynth (neurosynth.org) using “pain” **(A)**, “foot” **(B)**, and “hand” **(C)** as individual keywords. The foot and hand keywords were chosen as correlates for painful and non-painful somatosensory activity, respectively. Selected axial (top row), sagittal (bottom row, left), and coronal (bottom row, right) slices representing the spatial distribution of each functional association area are shown for each keyword. Each association map was treated as an individual ROI for inclusion in our connectivity analysis.

For connectivity analysis, we used a combined parcellation comprised of 361 regions of interest (ROIs) as well as 3 separately defined ROIs that we hypothesized to be involved in NP processing. The combined parcellation included 333 gray matter ROIs covering cerebral cortex (30), 14 subcortical gray matter ROIs (31), and 14 ROIs in cerebellar cortex (32). We isolated 3 composite ROIs using Neurosynth (neurosynth.org), to assess correlations with regions specifically associated with pain and somatosensation. Neurosynth automatically parses a wide-ranging database of neuroimaging articles and performs a meta-analysis of those articles in order to compute statistical maps of regions that have been shown to be correlated with the user-provided terms (33). We generated association maps with Neurosynth using “pain,” “foot,” and “hand” as individual keywords for database query. Visualizations of these maps can be found in Figure 2. All pain group subjects exhibited pain symptoms in one or both of their feet and lacked pain symptoms in their hands. Therefore, foot and hand were chosen as correlates for painful and non-painful somatosensory connectivity. For each set of regions, Fisher-transformed correlations between each of the ROIs were averaged for each subject between the two 15-min rs-fMRI acquisitions for that subject and group statistics were calculated using mean head motion, age, sex, and time since SCI as nuisance covariates.

## Results

### Cohort Characterization

**Figure 3:**
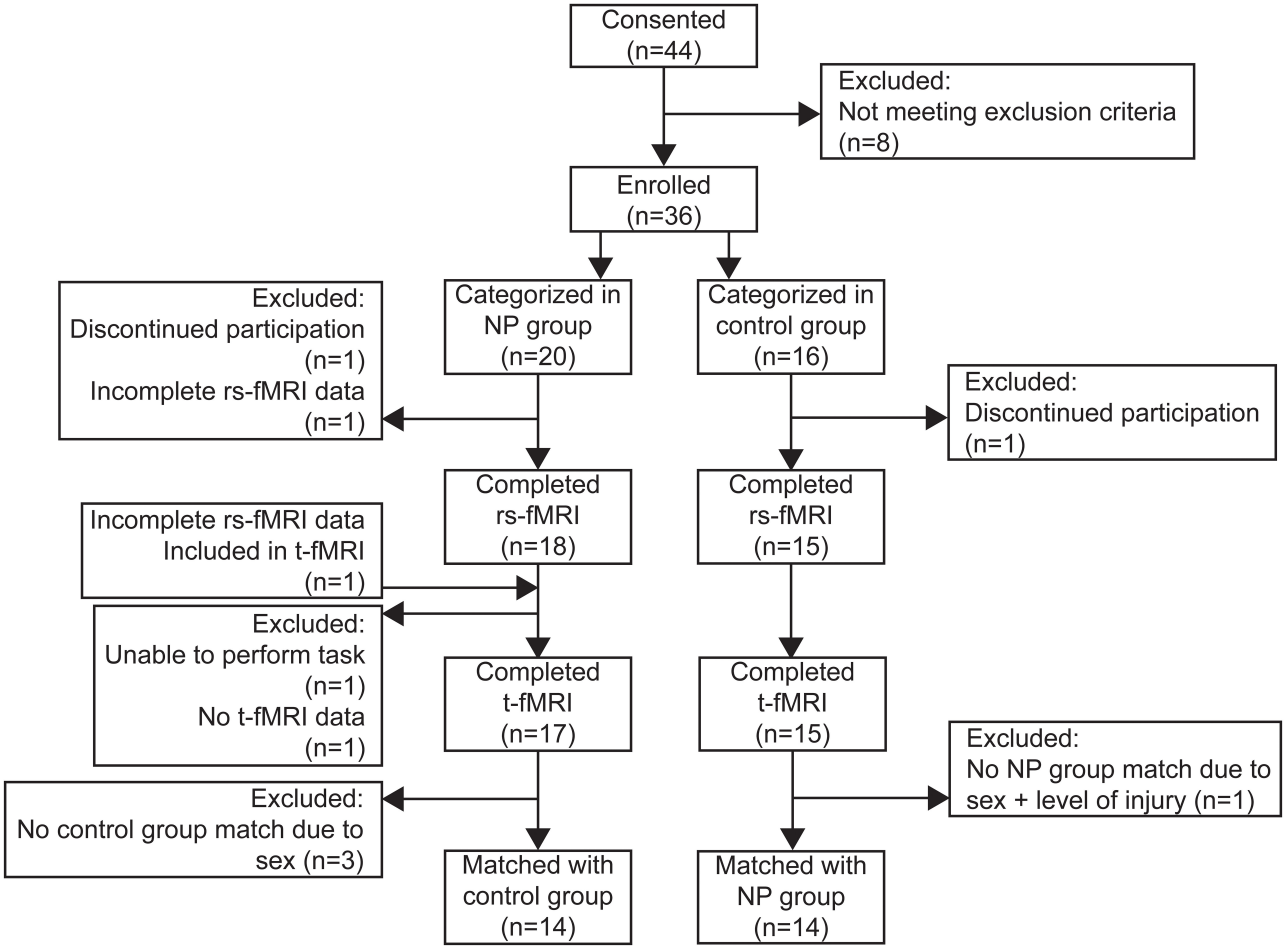
Flow diagram of subjects enrolled and included in resting state connectivity (rs-FMRI) and task-based (t-fMRI) analyses.

A total of 36 subjects with SCIs between spinal levels C4 and T12 were enrolled and underwent the imaging protocol for this study. Details for each subject are summarized in Supplementary Table 1. Of the 36 subjects recruited for this study, 20 (14 male) were categorized into the pain group and 16 (14 male) in the control group. Two subjects discontinued participation during the MRI protocol due to anxiety and were therefore excluded from functional imaging analysis, but their data is included in the cohort characterization results below. A flow diagram showing the distribution of subjects for each analysis and reasons for exclusion at each step is shown in Figure 3.

We found no significant differences between groups in any demographic category tested, except marital status for which the pain group had a higher proportion of married subjects. There was also no significant difference between groups in the ratio of subjects taking psychoactive medications (*p* = 0.12). The majority of subjects (16 pain, 8 control) were on regular oral or intrathecal doses of muscle relaxants or antispasmodics, such as baclofen. In addition, there were subjects taking antidepressant medications (6 pain, 4 control), antiepileptic medications (9 pain, 1 control), and narcotic pain medications (6 pain). Pain group subjects showed significantly higher levels of both anxiety (*p* < 0.00005) and depression (*p* < 0.005) compared to the control group. Table 1 shows a summary of the pain and control groups for each of the main demographics and characteristics captured for this study.

Pain group subjects rated their pain at the time of the scan at an average ± standard deviation of 3.5 ± 1.7 out of 10. Pain group subjects also rated their worst pain in the 24 h prior to the scan as 5.5 ± 2.2 and best or lowest pain levels as 2.1 ± 1.1 on average. Overall pain severity for pain subjects, scored using the BPI was 3.9 ± 1.8 and functional interference of pain was 3.6 ± 2.9 out of 10.

The NPSI captures the severity of neuropathic symptoms and is broken down into sub-categories, which include burning, pressure, paroxysmal, evoked, and paresthesia pain. Within the pain group, average ratings for each of the sub-categories for the 24 h prior to each subject's scan were 4.6 ± 2.5 for burning, 1.6 ± 1.7 for pressure, 3.3 ± 2.5 for paroxysmal, 1.7 ± 2.1 for evoked, and 4.4 ± 2.2 paresthesia pain. The sub-category for which neuropathic symptoms were rated as most severe was burning for 9 subjects, pressure for 1, paroxysmal for 3, evoked for 1, and paresthesia for 6 of the 20 pain group subjects. The average maximum NPSI sub-score for each subject was 5.7 ± 1.9.

After the scan, subjects rated pain levels during the MRI for burning as 2.7 ± 2.1, pressure as 1.1 ± 1.3, paroxysms as 2.0 ± 2.0, and paresthesia as 3.2 ± 2.2. The evoked pain sub-category asks about the severity of pain in response to brushing, pressure, and sensory contact stimuli, which were not administered during the MRI protocol. Therefore, this sub-category was excluded from the post-scan survey. During their time in the scanner, the most severe symptoms were burning for 8 subjects, pressure for 1 subject, paroxysms for 1 subject, and paresthesia for 10 subjects with the average maximum sub-score rating of 3.7 ± 2.1.

### Task fMRI

Imaging from a total of 32 subjects was used for this analysis. One subject was excluded because, although they were categorized into the pain group, they were not experiencing NP symptoms at the time of the scan and were therefore unable to perform the requirements of the task. A second subject was excluded because they did not have t-fMRI data of usable quality due to technical error (Figure 3).

We assessed the difference between groups for the pain minus non-pain focus contrast to identify regions of increased activity during the pain focus state in our NP subjects. A total of 14 subjects per group (13 male) were included in this comparison. Six subjects did not have matches in the opposite subject group and were therefore excluded.

**Figure 4:**
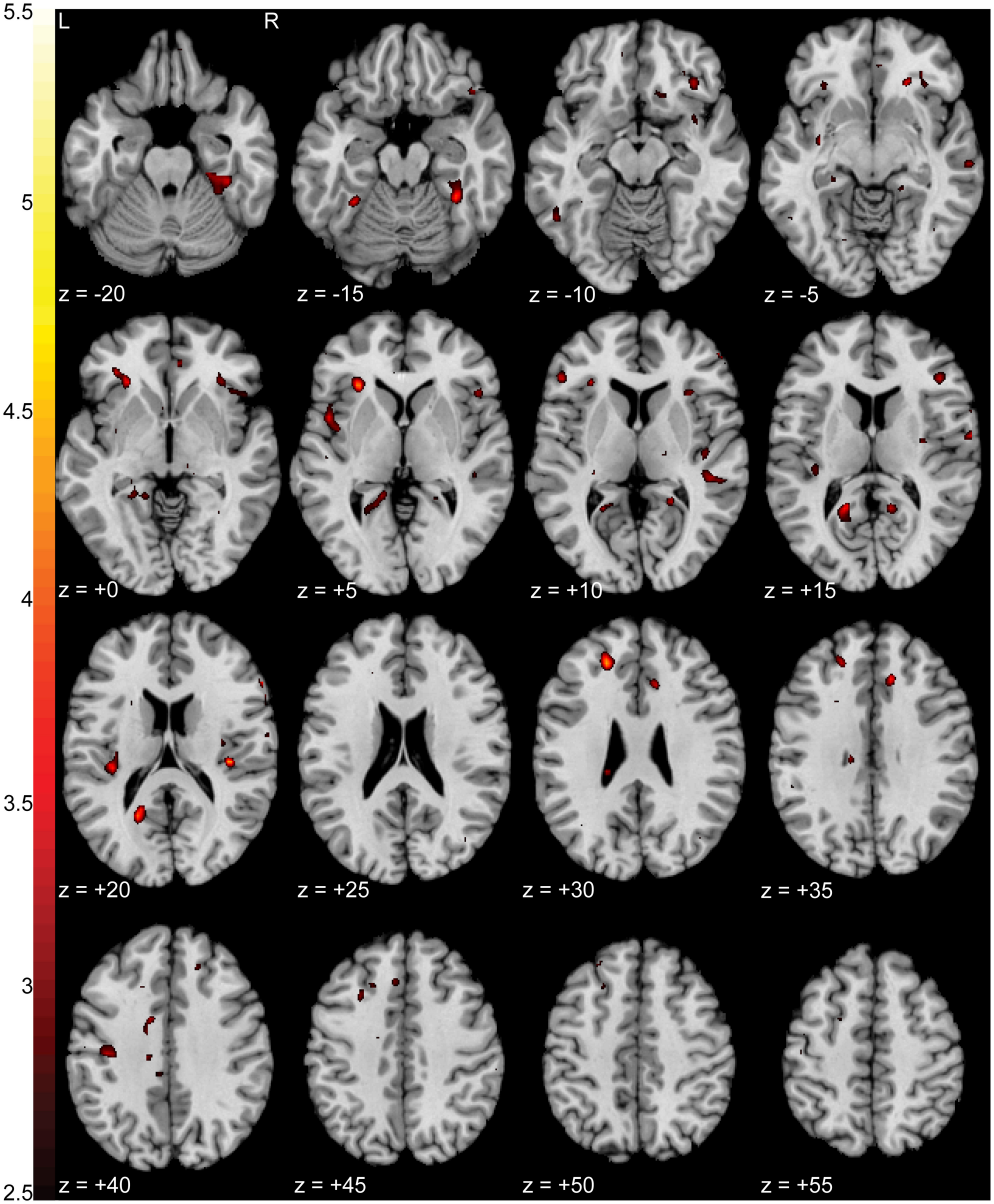
Increased activation in NP subjects compared to controls during the pain focus state in contrast to the non-pain focus state. T-statistic mapping of increased BOLD activation (*p* < 0.05 uncorrected) in NP subjects during the pain focus state when compared to the pain minus non-pain contrast in control subjects. Increased activity was seen in bilateral anterior and posterior insula, left retrosplenial cortex, and ventromedial prefrontal cortex. Clusters with fewer than 10 contiguous voxels were filtered for clarity. The difference between axial slices is 5 mm and *z*-values are relative to the AC/PC plane.

When comparing BOLD activation between groups during the pain focus state, we observed increased activation (*p* < 0.0005 uncorrected) in the pain group when compared to the control group in left ventromedial prefrontal cortex (vmPFC) (*T* = 4.48), left anterior insula (*T* = 4.35), left retrosplenial cortex (*T* = 4.23), and right fusiform (*T* = 4.23). Figure 4 shows a T-statistic map of the regions of increased activation (*p* < 0.05 uncorrected) in the pain group during the pain focus state in contrast to the non-pain focus state. Detailed cluster and peak level statistics for this between groups contrast can be found in Supplementary Table 2. We found no significant regions of increased activation in the control subjects compared to the pain subjects during the pain focus state. When repeating this analysis using the SnPM approach, no significant clusters were identified using a cluster defining threshold of *p* < 0.001. At a relaxed cluster defining threshold of *p* < 0.01, we identified a cluster in the anterior insula with a significance of *p* < 0.006 uncorrected, with a FWE corrected *p*-value of 0.12. This cluster overlapped with the increased activation seen with the SPM results in anterior insula. Supplementary Figure 1 shows a T-statistic map of the regions of increased activation in the pain group during the pain focus state in contrast to the non-pain focus state using the SnPM analysis. Detailed cluster and peak level statistics for this between groups contrast using the SnPM analysis can be found in Supplementary Table 3.

For within group comparisons, we assessed activation differences between the two focus states for each group individually. Because matched controls were not required, we included all 17 pain group subjects (14 male) and 15 control group subjects (13 male) with usable t-fMRI for these comparisons.

**Figure 5:**
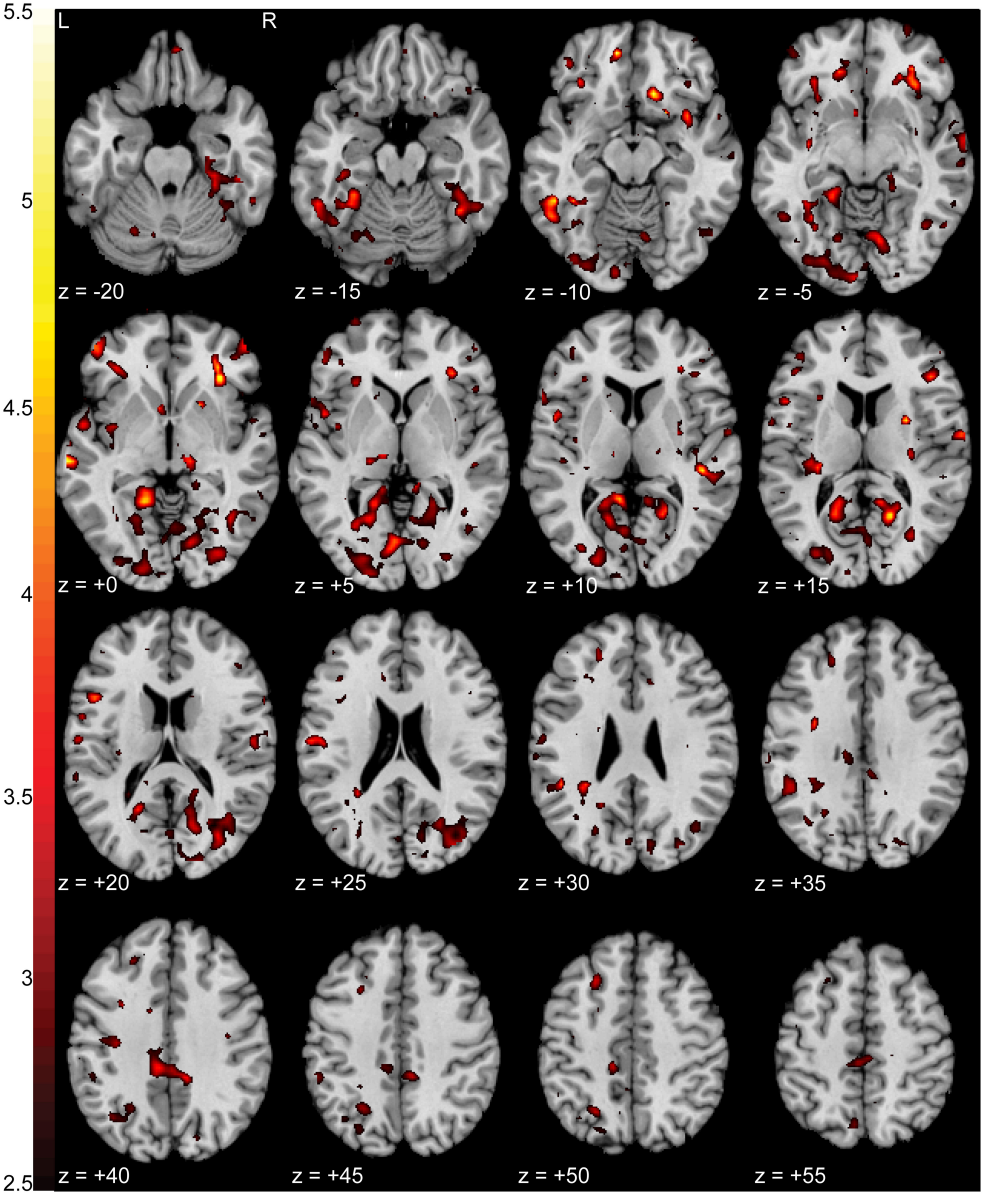
Increased activation in NP subjects during the pain focus state compared to the non-pain focus state. T-statistic mapping of increased BOLD activation (*p* < 0.05 uncorrected) during the pain focus state when compared to the non-pain focus state in the NP subject group. Increased activity was seen bilaterally in precuneus, anterior insula, middle temporal gyrus, middle cingulate cortex, and orbitofrontal cortex, and unilaterally in the left lingual gyrus. Clusters fewer than 10 contiguous voxels were filtered for clarity. The difference between axial slices is 5 mm and *z*-values are relative to the AC/PC plane.

Pain subjects' ratings of their pain severity was significantly different between focus states (*p* < 0.01e-60) with an average NPRS rating of 3.86 ± 1.58 during the pain focus state and 1.92 ± 1.53 during the non-pain focus state. There were increases in activation (*p* < 0.0005 uncorrected) in the pain during the pain focus state compared to the non-pain focus state in both right (*T* = 5.09) and left (*T* = 4.56) inferior precuneus, left middle temporal gyrus (*T* = 4.56), and right anterior insula (*T* = 4.89). There were also smaller regions of increased activation in bilateral orbitofrontal cortex, middle cingulate cortex, and right lingual gyrus. Figure 5 shows a T-statistic map of the regions showing increased activation (*p* < 0.05 uncorrected) during the pain focus state when compared to the non-pain focus state in the pain group subjects. Detailed cluster and peak level statistics can be found in Supplementary Table 4. There were no brain regions that showed increased BOLD activation during the non-pain focus state when compared to the pain focus state. In the control group subjects, there were no significant regions with differences in BOLD activation between the two focus states. Our *post-hoc* analysis looking at the pain and control groups individually to identify significant activation in either the pain or the non-pain focus state did not show any significant activation in either state for either group.

### Resting-State Connectivity

Of the 34 subjects with usable fMRI data, 33 were used for this analysis. One pain group subject was excluded because we did not have recorded pulse and respiratory data with which to correct their rs-fMRI time series. Thus, we compared rs-fMRI data between 18 pain group (13 male) and 15 control group (14 male) subjects (Figure 3).

**Figure 6:**
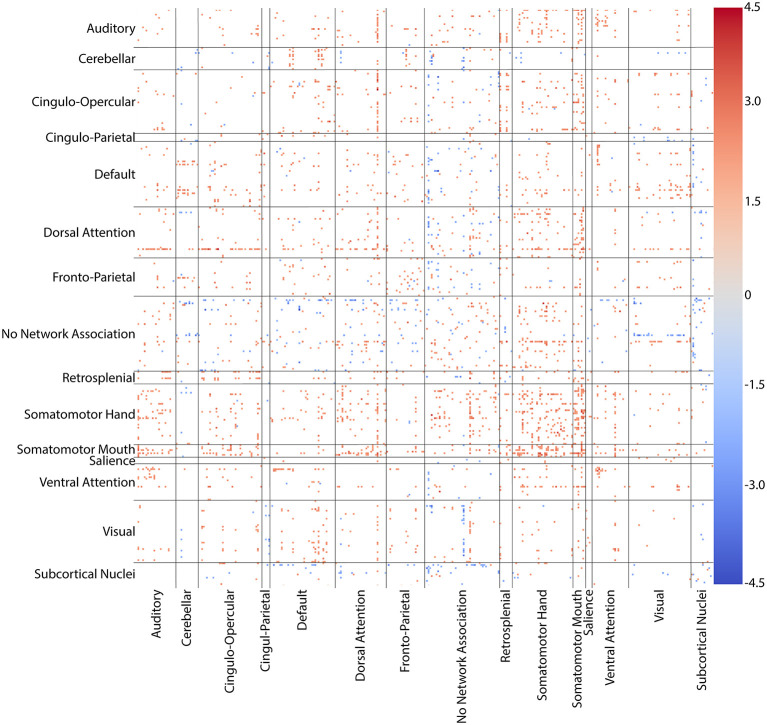
Connectivity matrix of regions with altered connectivity between NP and control groups. T-statistic values for whole-brain, all-to-all comparisons between groups for the 361 ROI combined cortical, subcortical, and cerebellar parcellation. Somatomotor hand, somatomotor mouth, and dorsal attention networks showed widespread increases in connectivity with nearly every other network. Hyperconnectivity was particularly dense in somatomotor-somatomotor connections. Only connections with differences at *p* < 0.05 uncorrected are colored. Warm colors (reds) indicate an increase in connectivity in the NP group when compared to controls. Cool colors (blues) indicate a decrease in the NP group.

We found no significant difference in head motion using the mean motion across both resting scans for each subject (*p* = 0.2916). Whole brain, all-to-all comparisons between pain and control groups for the 361 ROI combined parcellation showed increases (*p* < 0.05 uncorrected) in connectivity between regions of the anterior cingulate, medial prefrontal cortex, and somatosensory cortex in pain group subjects. The cortical regions of the combined parcellation are categorized into associative communities, which include auditory, cingulo-opercular, cingulo-parietal, default, dorsal attention, fronto-parietal, retrosplenial, somatomotor hand, somatomotor mouth, salience, and ventral attention networks as well as a group of regions with no specific network association (30). Decreases (*p* < 0.05 uncorrected) in connectivity were seen between subcortical nuclei and the default network, dorsal attention network, and regions with no network associations, though these effects were less pronounced than the increases in connectivity seen among other networks. There were widespread, increases (*p* < 0.05 uncorrected) in connectivity between both somatomotor networks and nearly every other network. This hyperconnectivity was specifically focused in the somatomotor networks themselves. Generalized hyperconnectivity was also seen throughout the dorsal attention network, again with higher rates of increase in somatomotor networks. Figure 6 shows a connectivity matrix of T-statistic values for regions showing differences between groups for the cortical, subcortical, and cerebellar networks in the 361-ROI parcellation.

**Figure 7:**
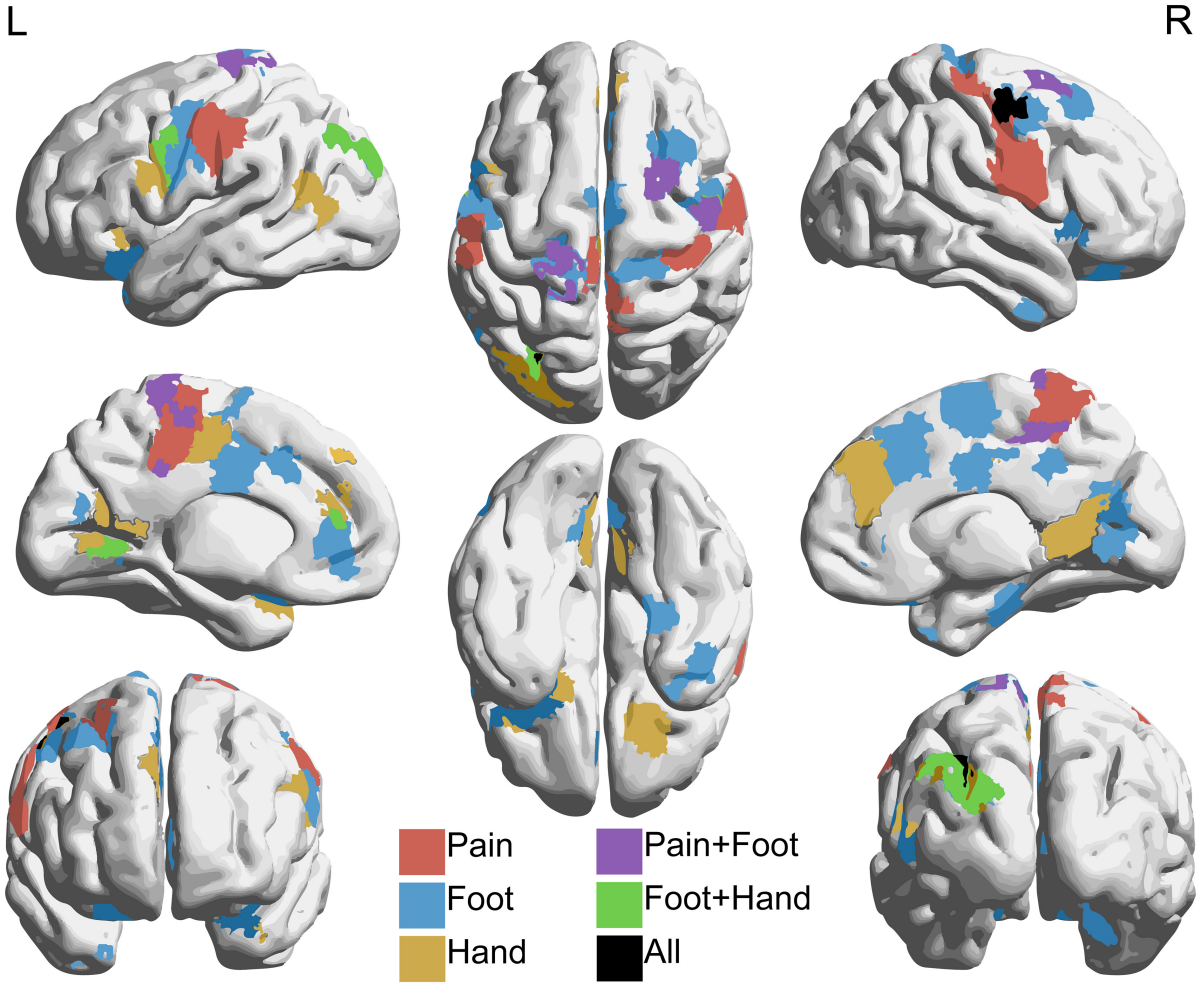
Regions with increased connectivity to pain, foot, and hand association regions in NP subjects compared to controls. Regions within the 361 combined parcellation showing increased connectivity (*p* < 0.05 uncorrected) with individually defined regions associated with pain, foot, and hand in NP subjects when compared to controls. Hyperconnectivity was seen between the pain region and parts of the dorsal attention, ventral attention, and somatomotor networks. The foot region showed increased connectivity with parts of the cingulo-opercular, dorsal attention, fronto-parietal, and somatomotor networks. The hand region showed increased connectivity with default, dorsal attention, fronto-parietal, ventral attention, and somatomotor networks. Lateral (top left and right), medial (middle left and right), anterior (bottom left), posterior (bottom right), superior (top middle), and inferior (bottom middle) views are shown.

The hyperconnectivity in pain subjects compared to controls was further highlighted when comparing connectivity between the pain and foot ROIs with the combined parcellation. Increases (*p* < 0.05 uncorrected) in connectivity were seen between the pain ROI and regions within the dorsal attention, ventral attention, and both somatomotor association networks, which include areas within sensorimotor cortex, cingulate cortex, and prefrontal cortex. Increased connectivity was also observed between the foot ROI and sensorimotor, cingulate, inferior medial parietal, and dorsolateral prefrontal cortices, corresponding to regions within cingulo-opercular, dorsal attention, fronto-parietal, somatomotor hand, and unassociated networks. Connectivity with the hand ROI was also increased in somatomotor cortex and unilaterally in medial inferior parietal and dorsolateral prefrontal cortices, corresponding to default, dorsal attention, fronto-parietal, somatomotor hand, somatomotor mouth, ventral attention, and unassociated networks. The effect size of the correlations with the hand ROI, however, was smaller and more spatially confined than those with the pain and foot ROIs. Figure 7 shows T-statistic maps of regions showing differences between groups in connectivity between the combined parcellation and the pain, foot, and hand ROIs.

## Discussion

Our results indicate a positive relationship between NP after SCI and neurological activity in insular, cingulate, somatosensory, medial prefrontal, and inferior medial parietal areas of cerebral cortex. All of these regions have consistently been indicated in various other pain conditions (12, 14, 15, 17–19, 21, 34, 35), but their specific relationship to NP in the SCI population has not yet been thoroughly explored. The SCI population is a particularly difficult population to study, due to a vast amount of variation between patients in motor function and sensation below the level of injury (3) and chronic secondary complications (36), among other things that may influence neurological behavior. We mitigated these issues by strictly controlling our study cohort, excluding those with chronic secondary conditions and/or motor function below their level of SCI and maintaining 50:50 ratios of those with and without sensation below their level of SCI. We also ensured that there were no significant differences in range of injury levels between groups (i.e., neither group had a significantly larger proportion of tetraplegics or paraplegics), so that upper extremity function was not a contributing factor for differences between groups.

With this carefully controlled cohort, we have examined both short-term neurological activation during a somatosensory focus task and spontaneous low-frequency correlations across the brain over time that are associated with NP after SCI. Comparing these results with structurally-based neurological changes associated with NP in the same cohort^1^, allow for validation across modalities and provide support for the identification of specific targets for future mechanistic research and targeted treatment approaches.

Our t-fMRI paradigm implemented a novel task that we developed in attempt to elucidate regions involved specifically in NP perception. This approach breaks from previous, commonly used elicited pain paradigms by identifying activity-based differences during focus on intrinsic somatosensation in painful vs. non-painful areas of the body. We observed consistent increases in activation in anterior insula in pain group subjects both between groups during the pain focus state and within the pain group between the pain focus state and the non-pain focus state. Anterior insula has consistently been implicated in both acute pain perception in healthy adults as well as pain processing in various neuropathic and non-neuropathic chronic pain populations (18, 34, 35, 37, 38). In the SCI population, increased activity in anterior insula was seen during a motor imagery task in SCI subjects with NP when compared to healthy controls (39), indicating that activity in this region may be involved in NP processing regardless of external input.

Anterior insula is also known to be involved in the maintenance of tonic alertness and somatosensory attention (40). In patients with peripheral nerve injury resulting in NP and somatosensory alteration, anterior insula was implicated both in NP perception and neurological representation of the body parts in which somatosensation was altered (41). These neurological responses were reversed as the effects of the peripheral nerve injuries decreased over time (41). Our results indicate that anterior insula may be specifically involved in NP processing and increased somatosensory-spatial integration of painful body areas in the SCI population as well. The fact that increases in activity were seen in both between and within group comparisons, but only during the pain focus state of the task indicate that during attention to benign somatosensation, the presence of chronic NP does not result in differential activation. Increased activity in anterior insula during attention tasks may therefore indicate a specific response to areas of the body experiencing chronic pain and not generalized somatosensory attention in the NP SCI cohort.

Additional regions of the brain associated with somatosensory integration and representational knowledge were prevalent in both our t-fMRI and rs-fMRI results. Retrosplenial cortex, included as part of the posterior cingulate cortex (PCC), inferior parietal cortex (IPC), including the precuneus, and vmPFC showed increases in the pain group in activation during the pain focus state during our task (Figures 4, 5) and connectivity at rest to our pain and somatosensation ROIs (Figure 7). All of these regions are highly interconnected (42) and have been shown to have altered activity and connectivity patterns in other chronic pain conditions, cognitive impairment conditions, and depression (17, 18, 35, 43–45). The precuneus and PCC are particularly densely interconnected and are commonly associated with integration of external with internal stimulation and mental representation (46). These regions are also major components of the default network, for which there is a considerable body of knowledge linking default network activity to intrinsic function and cognition (47).

Although our results in regard to the default network were not particularly robust, the PCC was correlated with the pain, foot, and hand ROIs and the IPC was correlated with both foot and hand in our NP group subjects. Further, alterations in connectivity between these regions and regions of the somatomotor and dorsal attention networks have been associated with a variety of neurological pathologies (15, 42, 48). Both the somatomotor networks and the dorsal attention network showed generalized increases in resting connectivity with other networks as well as our pain, foot, and hand ROIs, indicating that discoordination between PCC and IPC and these networks may contribute to tonic NP symptoms in this cohort. Other NP populations have seen similar mismatches in coordination between these networks as well. Diabetic neuropathy patients have shown altered resting state activity in several areas and networks of the brain including precuneus, prefrontal cortex, dorsal and ventral attention networks, and pre- and postcentral gyri, and it is hypothesized that the discoordination between these regions is due to impaired ability to process external vs. internal stimuli (15, 18).

It is already known that SCI results in impaired sensory-motor function (36) and all of the above listed regions were positively correlated with one or more of our pain and somatosensory ROIs in our NP group. However, SCI subjects have been shown to have decreased connectivity in somatosensory networks when compared to healthy controls when controlling for presence of NP within the SCI cohort (8). It can therefore be speculated that increased connectivity and discoordination between regions involved in somatosensory integration, including PCC and IPC, and the somatomotor and dorsal attention networks are indicative of NP in the SCI population. Strong conclusions cannot be drawn as to whether this is heightened activity in response to NP or a pathological compensatory mechanism in response to decreased activity that occurs due to the sensory and motor deficits resultant of SCI. However, this study has identified specific regions and connections of dysregulated activity specific to the SCI cohort that may be targeted in future mechanistic research to further improve our understanding of NP.

Limitations of this study include variability in pain focus location during our t-fMRI. In an effort to obtain the greatest contrast between painful and non-painful somatosensation, we asked pain group subjects to identify their most painful body part. The majority of the pain subjects (*N* = 14) identified their feet or lower extremities, though there were some subjects for whom their back (*N* = 2) or shoulders (*N* = 1) were most painful. This difference in somatotopic focus during our task may have resulted in differential activation patterns in both pain subjects and their control group counter parts who were asked to focus on the same body parts. Differential activation and connectivity patterns may have also been caused by the psychoactive medications taken by many of the subjects. However, we were unable to control for subjects' medication schedules and dosages due to ethical constraints. Additionally, we did not control for laterality of pain in our pain subjects, though anecdotally the majority of subjects reported diffuse bilateral pain. We further expect that for complex tasks, like pain, that there is enough between-subject heterogeneity in response that we suspect we would need a larger sample size to clearly define the main effect of the task.

Finally, our strict methods to control our subject pool, while important to minimize influence of factors other than NP on our cohort, limited the size of our cohort and subsequently our statistical power. Because of this, we were unable to identify connectivity or activation differences between groups that remained significant after corrections for multiple comparisons. While this does limit our ability to draw strong conclusions about the functional correlates identified here, our results indicate increased activity and connectivity associated with our pain group that agree with previous literature. The goal of this pilot study was to provide exploratory evidence for functional correlations with NP in a strictly controlled SCI population. We therefore took a discovery-based approach to identifying activation and connectivity based differences between our pain and control groups in order to provide meaningful insight for future clinical studies with larger, well-controlled SCI populations.

Although this study was not powered to detect false discovery rate corrected differences between groups for a whole brain discovery analysis, the specificity of the regions identified in our results and their functional associations to sensation and pain integration align with a priori hypotheses based on previous pain literature of increases in connectivity in somatomotor, insular, frontal, and medial parietal regions (11–15). Overall, this exploratory study has [1] presented a novel task approach to identify regions of the brain associated with chronic NP; [2] identified regions of increased task-based activation associated with NP after SCI within the insula, prefrontal, and medial inferior parietal cortices; and [3] identified regions of increased functional connectivity associated with NP after SCI within sensorimotor, cingulate, prefrontal, and inferior medial parietal cortices. This work also agrees with our structural analysis within the same cohort^1^, identifying specific regions of the brain associated with significant neurological alteration in SCI subjects with NP across multiple MRI modalities. Together, the results of each of these studies serve to substantiate the other and provide compelling evidence for somatosensory processing regions of inferior medial parietal cortex as a novel target for therapeutic intervention to treat NP after SCI.

## Data Availability Statement

The raw data supporting the conclusions of this article will be made available by the authors, without undue reservation.

## Ethics Statement

The studies involving human participants were reviewed and approved by University of Utah Institutional Review Board. The patients/participants provided their written informed consent to participate in this study.

## Author Contributions

SB carried out study procedures, executed data analysis, and prepared the first manuscript drafts. SB, JA, CB, and MM provided intellectual input into the study design, subject recruitment criteria, and methodological approach for both manuscripts. JK provided additional intellectual input and technological guidance for the analysis. CB provided project guidance as well as the computational resources necessary for the completion of the project. All authors participated in critical review of the manuscript.

## Conflict of Interest

The authors declare that the research was conducted in the absence of any commercial or financial relationships that could be construed as a potential conflict of interest.

## Footnotes

^1^Black SR, Janson A, Mahan M, Anderson J, Butson CR. Structural hyperconnectivity and gray matter volume changes associated with neuropathic pain after spinal cord injury. *Rev*. (unpublished).

## References

[1] Finnerup NB Haroutounian S Kamerman P Baron R Bennett DLH Bouhassira D. Neuropathic pain: an updated grading system for research and clinical practice. Pain. (2016) 157:1599–606. 10.1097/j.pain.0000000000000492 27115670 PMC4949003

[2] Burke D Fullen BM Stokes D Lennon O. Neuropathic pain prevalence following spinal cord injury: a systematic review and meta-analysis. Eur J Pain. (2017) 21:29–44. 10.1002/ejp.905 27341614

[3] Center NSCIS. Spinal Cord Injury Facts and Figures at a Glance 2020 SCI Data Sheet. (2020). Available online at: www.msktc.org/sci/model-system-centers (accessed July 2, 2020).

[4] Finnerup NB Baastrup C Jensen TS. Neuropathic pain following spinal cord injury pain: mechanisms and treatment. Scand J Pain. (2009) 1(Suppl. 1):S3–11. 10.1016/S1877-8860(09)70003-5

[5] Zheng W Chen Q Chen X Wan L Qin W Qi Z. Brain white matter impairment in patients with spinal cord injury. Neural Plast. (2017) 2017:1–8. 10.1155/2017/4671607 28255458 PMC5309430

[6] Chen Q Zheng W Chen X Wan L Qin W Qi Z. Brain gray matter atrophy after spinal cord injury: a voxel-based morphometry study. Front Hum Neurosci. (2017) 11:211. 10.3389/fnhum.2017.00211 28503142 PMC5408078

[7] Hou J-M Yan R-B Xiang Z-M Zhang H Liu J Wu Y-T. Brain sensorimotor system atrophy during the early stage of spinal cord injury in humans. Neuroscience. (2014) 266:208–5. 10.1016/j.neuroscience.2014.02.013 24561217

[8] Min Y-S Park JW Jin SU Jang KE Nam HU Lee Y-S. Alteration of resting-state brain sensorimotor connectivity following spinal cord injury: a resting-state functional magnetic resonance imaging study. J Neurotrauma. (2015) 32:1422–7. 10.1089/neu.2014.3661 25945389

[9] Faden AI Wu J Stoica BA Loane DJ. Progressive inflammation-mediated neurodegeneration after traumatic brain or spinal cord injury. Br J Pharmacol. (2016) 173:681–91. 10.1111/bph.13179 25939377 PMC4742301

[10] Stroman PW Khan HS Bosma RL Cotoi AI Leung R Cadotte DW. changes in pain processing in the spinal cord and brainstem after spinal cord injury characterized by functional magnetic resonance imaging. J Neurotrauma. (2016) 33:1450–60. 10.1089/neu.2015.4257 26801315

[11] Baliki MN Geha PY Vania Apkarian A Apkarian AV. Spontaneous pain and brain activity in neuropathic pain: functional MRI and pharmacologic functional MRI studies. Curr Pain Headache Rep. (2007) 11:171–7. 10.1007/s11916-007-0187-3 17504643

[12] Farmer MA Chanda ML Parks EL Baliki MN Apkarian AV Schaeffer AJ. Brain functional and anatomical changes in chronic prostatitis/chronic pelvic pain syndrome. J Urol. (2011) 186:117–24. 10.1016/j.juro.2011.03.027 21571326 PMC4889821

[13] Maihöfner C Baron R DeCol R Binder A Birklein F Deuschl G. The motor system shows adaptive changes in complex regional pain syndrome. Brain. (2007) 130:2671–87. 10.1093/brain/awm131 17575278

[14] Lee MJ Park BY Cho S Kim ST Park H Chung CS. Increased connectivity of pain matrix in chronic migraine: a resting-state functional MRI study. J Headache Pain. (2019) 20:29. 10.1186/s10194-019-0986-z 30909865 PMC6734233

[15] Cauda F D'Agata F Sacco K Duca S Cocito D Paolasso I. Altered resting state attentional networks in diabetic neuropathic pain. J Neurol Neurosurg Psychiatry. (2010) 81:806–11. 10.1136/jnnp.2009.188631 19955113

[16] May A. Chronic pain may change the structure of the brain. Pain. (2008) 137:7–15. 10.1016/j.pain.2008.02.034 18410991

[17] Kornelsen J Sboto-Frankenstein U McIver T Gervai P Wacnik P Berrington N. Default mode network functional connectivity altered in failed back surgery syndrome. J Pain. (2013) 14:483–91. 10.1016/j.jpain.2012.12.018 23498869

[18] Cauda F Sacco K Duca S Cocito D D'Agata F Geminiani GC. Altered resting state in diabetic neuropathic pain. PLoS ONE. (2009) 4:e4542. 10.1371/journal.pone.0004542 19229326 PMC2638013

[19] Tracey I Mantyh PW. The cerebral signature for pain perception and its modulation. Neuron. (2007) 55:377–91. 10.1016/j.neuron.2007.07.012 17678852

[20] Peyron R Laurent B García-Larrea L. Functional imaging of brain responses to pain. a review and meta-analysis. Neurophysiol Clin. (2000) 30:263–88. 10.1016/S0987-7053(00)00227-6 11126640

[21] Wager TD Atlas LY Lindquist MA Roy M Woo C-W Kross E. An fMRI-based neurologic signature of physical pain. N Engl J Med. (2013) 368:1388–97. 10.1056/NEJMoa1204471 23574118 PMC3691100

[22] Gruener H Zeilig G Laufer Y Blumen N Defrin R. Differential pain modulation properties in central neuropathic pain after spinal cord injury. Pain. (2016) 157:1415–24. 10.1097/j.pain.0000000000000532 26894913

[23] Snaith RP. The hospital anxiety and depression scale. Health Qual Life Outcomes. (2003) 1:29. 10.1186/1477-7525-1-29 12914662 PMC183845

[24] Bouhassira D Attal N Fermanian J Alchaar H Gautron M Masquelier E. Development and validation of the neuropathic pain symptom inventory. Pain. (2004) 108:248–57. 10.1016/j.pain.2003.12.024 15030944

[25] Cleeland CS Ryan KM. Pain assessment: global use of the brief pain inventory. Ann Acad Med Singapore. (1994) 23:129–38. 8080219

[26] Friston KJ Karl J Ashburner J Kiebel S Nichols T Penny WD. Statistical Parametric Mapping : The Analysis of Funtional Brain Images. Oxford, UK: Elsevier/Academic Press (2007).

[27] Holmes AP Blair RC Watson JDG Ford I. Nonparametric analysis of statistic images from functional mapping experiments. J Cereb Blood Flow Metab. (1996) 16:7–22. 10.1097/00004647-199601000-00002 8530558

[28] Anderson JS Druzgal TJ Lopez-Larson M Jeong E-K Desai K Yurgelun-Todd D. Network anticorrelations, global regression, and phase-shifted soft tissue correction. Hum Brain Mapp. (2011) 32:919–34. 10.1002/hbm.21079 20533557 PMC3220164

[29] Birn RM Smith MA Jones TB Bandettini PA. The respiration response function: the temporal dynamics of fMRI signal fluctuations related to changes in respiration. Neuroimage. (2008) 40:644–54. 10.1016/j.neuroimage.2007.11.059 18234517 PMC2533266

[30] Gordon EM Laumann TO Adeyemo B Huckins JF Kelley WM Petersen SE. Generation and evaluation of a cortical area parcellation from resting-state correlations. Cereb Cortex. (2016) 26:288–303. 10.1093/cercor/bhu239 25316338 PMC4677978

[31] Fischl B Salat DH Busa E Albert M Dieterich M Haselgrove C. Whole brain segmentation: automated labeling of neuroanatomical structures in the human brain. Neuron. (2002) 33:341–55. 10.1016/S0896-6273(02)00569-X 11832223

[32] Buckner RL Krienen FM Castellanos A Diaz JC Yeo BTT. The organization of the human cerebellum estimated by intrinsic functional connectivity. J Neurophysiol. (2011) 106:2322–45. 10.1152/jn.00339.2011 21795627 PMC3214121

[33] Yarkoni T Poldrack RA Nichols TE Van Essen DC Wager TD. Large-scale automated synthesis of human functional neuroimaging data. Nat Methods. (2011) 8:665–70. 10.1038/nmeth.1635 21706013 PMC3146590

[34] Apkarian AVV. A brain signature for acute pain. Trends Cogn Sci. (2013) 17:309–10. 10.1016/j.tics.2013.05.001 23747083 PMC3733463

[35] Baliki MN Chialvo DR Geha PY Levy RM Harden RN Parrish TB. Chronic pain and the emotional brain: specific brain activity associated with spontaneous fluctuations of intensity of chronic back pain. J Neurosci. (2006) 26:12165–73. 10.1523/JNEUROSCI.3576-06.2006 17122041 PMC4177069

[36] Sezer N Akkuş S Ugurlu FG. Chronic complications of spinal cord injury. World J Orthop. (2015) 6:24–33. 10.5312/wjo.v6.i1.24 25621208 PMC4303787

[37] Apkarian AV. Human brain imaging studies of chronic pain: translational opportunities. In: Kruger L Light AR editors. Translational Pain Research: From Mouse to Man. Boca Raton, FL: CRC Press/Taylor & Francis. p. 329–48. Available online at: http://www.ncbi.nlm.nih.gov/pubmed/21882455 (accessed November 29, 2018). 21882455

[38] Lin CS. Brain signature of chronic orofacial pain: a systematic review and meta-analysis on neuroimaging research of trigeminal neuropathic pain and temporomandibular joint disorders. PLoS ONE. (2014) 9:e94300. 10.1371/journal.pone.0094300 24759798 PMC3997345

[39] Gustin SM Wrigley PJ Henderson LA Siddall PJ. Brain circuitry underlying pain in response to imagined movement in people with spinal cord injury. Pain. (2010) 148:438–45. 10.1016/j.pain.2009.12.001 20092946

[40] Sadaghiani S D'Esposito M. Functional characterization of the cingulo-opercular network in the maintenance of tonic alertness. Cereb Cortex. (2015) 25:2763–73. 10.1093/cercor/bhu072 24770711 PMC4537431

[41] Hsieh P-CC Tseng M-TT Chao C-CC Lin Y-HH Tseng W-YYI Liu K-HH. Imaging signatures of altered brain responses in small-fiber neuropathy: reduced functional connectivity of the limbic system after peripheral nerve degeneration. Pain. (2015) 156:904–16. 10.1097/j.pain.0000000000000128 25734991

[42] Khalsa S Mayhew SD Chechlacz M Bagary M Bagshaw AP. The structural and functional connectivity of the posterior cingulate cortex: comparison between deterministic and probabilistic tractography for the investigation of structure-function relationships. Neuroimage. (2014) 102:118–27. 10.1016/j.neuroimage.2013.12.022 24365673

[43] Zhou Y Dougherty JH Hubner KF Bai B Cannon RL Hutson RK. Abnormal connectivity in the posterior cingulate and hippocampus in early Alzheimer's disease and mild cognitive impairment. Alzheimer's Dement. (2008) 4:265–70. 10.1016/j.jalz.2008.04.006 18631977

[44] Bai F Watson DR Yu H Shi Y Yuan Y Zhang Z. Abnormal resting-state functional connectivity of posterior cingulate cortex in amnestic type mild cognitive impairment. Brain Res. (2009) 1302:167–74. 10.1016/j.brainres.2009.09.028 19765560

[45] Cheng W Rolls ET Qiu J Xie X Wei D Huang C-CC. Increased functional connectivity of the posterior cingulate cortex with the lateral orbitofrontal cortex in depression. Transl Psychiatry. (2018) 8:1–0. 10.1038/s41398-018-0139-1 29691380 PMC5915597

[46] Cavanna AE Trimble MR. The precuneus: a review of its functional anatomy and behavioural correlates. Brain. (2006) 129:564–83. 10.1093/brain/awl004 16399806

[47] Fransson P Marrelec G. The precuneus/posterior cingulate cortex plays a pivotal role in the default mode network: evidence from a partial correlation network analysis. Neuroimage. (2008) 42:1178–84. 10.1016/j.neuroimage.2008.05.059 18598773

[48] Spreng RN Shoemaker L Turner GR. Executive functions and neurocognitive aging. In: Goldberg E editor. Executive Functions in Health and Disease. Cambridge, MA: Elsevier Inc. p. 169–96. 10.1016/B978-0-12-803676-1.00008-8

