# Supplementary material for: Functional Hyperconnectivity and Task-Based Activity Changes Associated With Neuropathic Pain After Spinal Cord Injury: A Pilot Study
Source: Front Neurol. 2021 Jun 10;12:613630. doi: 10.3389/fneur.2021.613630 (PMC8222514; doi:10.3389/fneur.2021.613630)
Supplement: Supplementary file 1 [file Data_Sheet_1.docx]

Supplementary Material

**Table S1. Summary of group, age, sex, and SCI information for all subjects.**

| **Subject ID** | **Pain or Control** | **Sex** | **Age** | **Level of SCI** | **Complete or Incomplete** | **AIS** | **Time Since SCI (mo.)** |
| --- | --- | --- | --- | --- | --- | --- | --- |
| 1 | Pain | M | 27 | C4 | Incomplete | B | 31 |
| 5 | Control | F | 22 | C6 | Incomplete | B | 66 |
| 6 | Control | M | 29 | C6 | Complete | A | 100 |
| 7 | Pain | M | 22 | C5 | Complete | B | 78 |
| 8 | Pain | M | 39 | C6 | Complete | A | 280 |
| 9 | Pain | M | 18 | C5 | Incomplete | B | 15 |
| 10 | Control | M | 34 | C7 | Complete | B | 131 |
| 13 | Control | M | 19 | C6 | Incomplete | B | 57 |
| 14 | Pain | M | 34 | C4 | Incomplete | A | 208 |
| 15 | Control | M | 25 | C4 | Incomplete | A | 45 |
| 16 | Pain | M | 42 | C6 | Incomplete | B | 209 |
| 17 | Control | M | 21 | C6 | Incomplete | B | 34 |
| 18 | Control | M | 28 | C5 | Incomplete | B | 118 |
| 19 | Pain | M | 27 | C6 | Incomplete | B | 39 |
| 21 | Pain | M | 38 | C6 | Incomplete | B | 38 |
| 21 | Pain | M | 38 | C7 | Incomplete | B | 38 |
| 22 | Pain | M | 35 | C4 | Complete | A | 163 |
| 24 | Pain | M | 27 | C8 | Incomplete | B | 55 |
| 25 | Control | M | 32 | C5 | Complete | A | 165 |
| 26 | Control | M | 30 | C6 | Incomplete | B | 60 |
| 27 | Control | M | 30 | C5 | Complete | A | 26 |
| 28 | Control | M | 23 | C4 | Incomplete | B | 79 |
| 29 | Pain | M | 39 | C5 | Incomplete | B | 281 |
| 30 | Pain | M | 43 | T3 | Complete | A | 36 |
| 32 | Pain | F | 32 | T4 | Complete | A | 61 |
| 33 | Pain | F | 41 | T6 | Complete | A | 16 |
| 34 | Pain | F | 18 | T6 | Complete | A | 16 |
| 35 | Pain | F | 18 | T5 | Complete | A | 98 |
| 36 | Pain | M | 41 | T5 | Complete | A | 43 |
| 37 | Control | M | 31 | T6 | Complete | A | 92 |
| 38 | Control | F | 36 | T6 | Incomplete | B | 173 |
| 39 | Control | M | 38 | T12 | Complete | A | 12 |
| 40 | Pain | F | 45 | T12 | Incomplete | B | 117 |
| 41 | Control | M | 24 | T5 | Complete | A | 37 |
| 42 | Pain | F | 32 | T12 | Incomplete | B | 79 |
| 43 | Control | M | 38 | T10 | Complete | A | 238 |
| 44 | Pain | M | 23 | T5 | Complete | A | 42 |

**Table S2. Cluster and peak level statistics and MNI coordinates for comparison of increased activation during pain focus state between NP subjects minus controls.**

| **Cluster-Level** | | | | **Peak-Level** | | | | | **mm** | **mm** | **mm** |
| --- | --- | --- | --- | --- | --- | --- | --- | --- | --- | --- | --- |
| **p**  **(FWE)** | **q**  **(FDR)** | **k**  **(E)** | **p**  **(uncorr)** | **p**  **(FWE)** | **q**  **(FDR)** | **T** | **(Z_≡_)** | **p**  **(uncorr)** |  |  |  |
| **0.365** | **0.193** | **1207** | **0.004** | **0.75** | **1** | **4.83** | **3.97** | **0.00004** | **-20** | **34** | **30** |
|  |  |  |  | 1 | 1 | 3.68 | 3.23 | 0.001 | 12 | 24 | 34 |
|  |  |  |  | 1 | 1 | 3.23 | 2.91 | 0.002 | -6 | 20 | 46 |
| **0.052** | **0.065** | **1982** | **0.001** | **0.891** | **1** | **4.58** | **3.82** | **0.00007** | **-28** | **22** | **2** |
|  |  |  |  | 0.943 | 1 | 4.44 | 3.73 | 0.00009 | -36 | -32 | 20 |
|  |  |  |  | 0.999 | 1 | 3.93 | 3.4 | 0.0003 | -48 | 2 | 6 |
| **0.787** | **0.463** | **824** | **0.015** | **0.963** | **1** | **4.36** | **3.69** | **0.0001** | **-18** | **-58** | **18** |
|  |  |  |  | 1 | 1 | 3.56 | 3.14 | 0.001 | -28 | -48 | -2 |
|  |  |  |  | 1 | 1 | 3.12 | 2.82 | 0.002 | -18 | -54 | 6 |
| **0.083** | **0.065** | **1798** | **0.001** | **0.975** | **1** | **4.3** | **3.65** | **0.0001** | **38** | **-28** | **20** |
|  |  |  |  | 1 | 1 | 3.88 | 3.37 | 0.0004 | 60 | 22 | 20 |
|  |  |  |  | 1 | 1 | 3.63 | 3.19 | 0.001 | 32 | -14 | 14 |
| **0.403** | **0.193** | **1165** | **0.005** | **0.994** | **1** | **4.13** | **3.53** | **0.0002** | **32** | **30** | **-8** |
|  |  |  |  | 1 | 1 | 3.81 | 3.32 | 0.0004 | 36 | 16 | 10 |
|  |  |  |  | 1 | 1 | 3.54 | 3.13 | 0.001 | 46 | 20 | -16 |
| **1** | **0.953** | **378** | **0.081** | **0.999** | **1** | **3.93** | **3.4** | **0.0** | **34** | **-46** | **-16** |
|  |  |  |  | 1 | 1 | 3.19 | 2.87 | 0.002 | 38 | -38 | -18 |
|  |  |  |  | 1 | 1 | 3.09 | 2.8 | 0.003 | 26 | -34 | -20 |
| **1** | **0.953** | **181** | **0.214** | **1** | **1** | **3.58** | **3.16** | **0.001** | **-30** | **-48** | **-16** |
|  |  |  |  | 1 | 1 | 2.07 | 1.96 | 0.025 | -34 | -34 | -16 |
|  |  |  |  | 1 | 1 | 2.04 | 1.94 | 0.026 | -38 | -62 | -12 |
| **1** | **0.953** | **222** | **0.171** | **1** | **1** | **3.51** | **3.11** | **0.001** | **-12** | **-4** | **42** |
|  |  |  |  | 1 | 1 | 3.34 | 2.99 | 0.001 | -14 | -12 | 42 |
|  |  |  |  | 1 | 1 | 2.23 | 2.1 | 0.018 | -22 | -20 | 54 |
| **1** | **0.953** | **264** | **0.138** | **1** | **1** | **3.42** | **3.04** | **0.001** | **12** | **-56** | **14** |
|  |  |  |  | 1 | 1 | 2.96 | 2.7 | 0.003 | 22 | -52 | 10 |
|  |  |  |  | 1 | 1 | 1.78 | 1.72 | 0.043 | 24 | -60 | 18 |
| **1** | **0.953** | **125** | **0.3** | **1** | **1** | **3.37** | **3** | **0.001** | **6** | **36** | **-2** |
|  |  |  |  | 1 | 1 | 2.75 | 2.53 | 0.006 | -4 | 42 | -2 |
|  |  |  |  | 1 | 1 | 2.56 | 2.38 | 0.009 | -8 | 30 | 6 |
| **1** | **0.953** | **55** | **0.499** | **1** | **1** | **3.35** | **2.99** | **0.001** | **16** | **16** | **-10** |
| **0.98** | **0.953** | **567** | **0.037** | **1** | **1** | **3.29** | **2.95** | **0.002** | **-20** | **-34** | **30** |
|  |  |  |  | 1 | 1 | 3.22 | 2.9 | 0.002 | -14 | -26 | 38 |
|  |  |  |  | 1 | 1 | 2.96 | 2.69 | 0.004 | -6 | -36 | 38 |
| **1** | **0.953** | **71** | **0.438** | **1** | **1** | **3.17** | **2.85** | **0.002** | **-20** | **-4** | **54** |
| **1** | **0.953** | **23** | **0.678** | **1** | **1** | **3.09** | **2.8** | **0.003** | **20** | **6** | **2** |
| **0.99** | **0.953** | **527** | **0.043** | **1** | **1** | **3.05** | **2.77** | **0.003** | **-40** | **-24** | **40** |
|  |  |  |  | 1 | 1 | 2.63 | 2.44 | 0.007 | -28 | -18 | 42 |
|  |  |  |  | 1 | 1 | 2.61 | 2.42 | 0.008 | -44 | -12 | 56 |
| **1** | **0.953** | **86** | **0.392** | **1** | **1** | **2.96** | **2.7** | **0.004** | **36** | **2** | **-12** |
|  |  |  |  | 1 | 1 | 2.6 | 2.41 | 0.008 | 36 | -4 | -6 |
| **1** | **0.953** | **69** | **0.445** | **1** | **1** | **2.93** | **2.68** | **0.004** | **-18** | **16** | **-8** |
|  |  |  |  | 1 | 1 | 2.49 | 2.32 | 0.01 | -8 | 20 | -6 |
| **1** | **0.953** | **179** | **0.217** | **1** | **1** | **2.92** | **2.66** | **0.004** | **-2** | **-24** | **58** |
|  |  |  |  | 1 | 1 | 2.25 | 2.12 | 0.017 | -4 | -34 | 56 |
| **1** | **0.953** | **53** | **0.507** | **1** | **1** | **2.84** | **2.6** | **0.005** | **-16** | **12** | **8** |
|  |  |  |  | 1 | 1 | 2.03 | 1.93 | 0.027 | -16 | 2 | 12 |
| **1** | **0.953** | **22** | **0.685** | **1** | **1** | **2.78** | **2.55** | **0.005** | **8** | **46** | **-20** |
| 1 | 0.953 | 90 | 0.38 | 1 | 1 | 2.77 | 2.55 | 0.005 | 68 | -38 | 24 |
|  |  |  |  | 1 | 1 | 2.27 | 2.14 | 0.016 | 68 | -32 | 18 |
| **1** | **0.953** | **44** | **0.549** | **1** | **1** | **2.77** | **2.54** | **0.005** | **16** | **-22** | **10** |
| **1** | **0.953** | **128** | **0.295** | **1** | **1** | **2.74** | **2.52** | **0.006** | **-52** | **-60** | **-8** |
| **1** | **0.953** | **41** | **0.564** | **1** | **1** | **2.73** | **2.52** | **0.006** | **-6** | **6** | **-2** |
| **1** | **0.953** | **298** | **0.117** | **1** | **1** | **2.72** | **2.51** | **0.006** | **42** | **-76** | **32** |
|  |  |  |  | 1 | 1 | 2.35 | 2.21 | 0.014 | 38 | -66 | 22 |
| **1** | **0.953** | **193** | **0.2** | **1** | **1** | **2.69** | **2.49** | **0.006** | **-18** | **-74** | **-6** |
|  |  |  |  | 1 | 1 | 2.39 | 2.24 | 0.013 | -28 | -76 | -14 |
|  |  |  |  | 1 | 1 | 2.14 | 2.03 | 0.021 | -24 | -84 | -10 |
| **1** | **0.953** | **36** | **0.592** | **1** | **1** | **2.69** | **2.48** | **0.007** | **12** | **-16** | **38** |
| **1** | **0.953** | **356** | **0.089** | **1** | **1** | **2.67** | **2.47** | **0.007** | **-52** | **-42** | **36** |
|  |  |  |  | 1 | 1 | 2.33 | 2.19 | 0.014 | -52 | -34 | 40 |
|  |  |  |  | 1 | 1 | 2.24 | 2.11 | 0.017 | -62 | -40 | 34 |
| **1** | **0.953** | **164** | **0.236** | **1** | **1** | **2.65** | **2.45** | **0.007** | **-38** | **-68** | **32** |
|  |  |  |  | 1 | 1 | 2.34 | 2.19 | 0.014 | -42 | -70 | 20 |
| **1** | **0.953** | **25** | **0.663** | **1** | **1** | **2.64** | **2.45** | **0.007** | **-10** | **44** | **-10** |
| **1** | **0.953** | **50** | **0.52** | **1** | **1** | **2.6** | **2.41** | **0.008** | **-44** | **-42** | **4** |
|  |  |  |  | 1 | 1 | 2.12 | 2.01 | 0.022 | -56 | -52 | 10 |
| **1** | **0.953** | **23** | **0.678** | **1** | **1** | **2.55** | **2.37** | **0.009** | **18** | **16** | **48** |
| **1** | **0.953** | **34** | **0.603** | **1** | **1** | **2.55** | **2.37** | **0.009** | **8** | **48** | **2** |
| **1** | **0.953** | **160** | **0.242** | **1** | **1** | **2.55** | **2.37** | **0.009** | **-8** | **-64** | **46** |
|  |  |  |  | 1 | 1 | 2.13 | 2.01 | 0.022 | -24 | -78 | 48 |
|  |  |  |  | 1 | 1 | 1.98 | 1.89 | 0.03 | -14 | -72 | 48 |
| **1** | **0.953** | **177** | **0.219** | **1** | **1** | **2.53** | **2.35** | **0.009** | **-40** | **8** | **24** |
|  |  |  |  | 1 | 1 | 2.4 | 2.25 | 0.012 | -24 | 10 | 20 |
|  |  |  |  | 1 | 1 | 1.97 | 1.88 | 0.03 | -38 | 18 | 28 |
| **1** | **0.953** | **13** | **0.766** | **1** | **1** | **2.53** | **2.35** | **0.009** | **-32** | **-14** | **30** |
| **1** | **0.953** | **22** | **0.685** | **1** | **1** | **2.52** | **2.35** | **0.009** | **36** | **-24** | **-4** |
| **1** | **0.953** | **68** | **0.448** | **1** | **1** | **2.52** | **2.35** | **0.009** | **-58** | **-16** | **20** |
|  |  |  |  | 1 | 1 | 2.19 | 2.07 | 0.019 | -56 | -14 | 28 |
|  |  |  |  | 1 | 1 | 1.76 | 1.7 | 0.045 | -56 | -8 | 36 |
| **1** | **0.953** | **53** | **0.507** | **1** | **1** | **2.52** | **2.35** | **0.01** | **50** | **4** | **-14** |
|  |  |  |  | 1 | 1 | 2.2 | 2.08 | 0.019 | 42 | -4 | -20 |
| **1** | **0.953** | **53** | **0.507** | **1** | **1** | **2.51** | **2.34** | **0.01** | **30** | **-60** | **2** |
| **1** | **0.953** | **16** | **0.737** | **1** | **1** | **2.48** | **2.31** | **0.01** | **34** | **-66** | **-52** |
| **1** | **0.953** | **11** | **0.788** | **1** | **1** | **2.48** | **2.31** | **0.01** | **-18** | **-54** | **-32** |
| **1** | **0.953** | **13** | **0.766** | **1** | **1** | **2.47** | **2.31** | **0.011** | **-16** | **58** | **6** |
| **1** | **0.953** | **32** | **0.616** | **1** | **1** | **2.47** | **2.31** | **0.011** | **12** | **60** | **14** |
| **1** | **0.953** | **119** | **0.312** | **1** | **1** | **2.46** | **2.29** | **0.011** | **0** | **46** | **18** |
|  |  |  |  | 1 | 1 | 2.33 | 2.19 | 0.014 | 10 | 40 | 14 |
| **1** | **0.953** | **16** | **0.737** | **1** | **1** | **2.45** | **2.29** | **0.011** | **-2** | **-8** | **-12** |
| **1** | **0.953** | **59** | **0.482** | **1** | **1** | **2.44** | **2.27** | **0.011** | **-30** | **34** | **-10** |
|  |  |  |  | 1 | 1 | 2.19 | 2.07 | 0.019 | -22 | 32 | -14 |
| **1** | **0.953** | **67** | **0.452** | **1** | **1** | **2.39** | **2.24** | **0.013** | **8** | **-4** | **44** |
|  |  |  |  | 1 | 1 | 2.1 | 1.99 | 0.023 | 8 | -10 | 54 |
| **1** | **0.953** | **41** | **0.564** | **1** | **1** | **2.38** | **2.23** | **0.013** | **18** | **-38** | **76** |
|  |  |  |  | 1 | 1 | 2.12 | 2.01 | 0.022 | 20 | -28 | 74 |
| **1** | **0.953** | **11** | **0.788** | **1** | **1** | **2.29** | **2.15** | **0.016** | **-26** | **-40** | **72** |
| **1** | **0.953** | **26** | **0.655** | **1** | **1** | **2.27** | **2.14** | **0.016** | **46** | **-60** | **2** |
| **1** | **0.953** | **26** | **0.655** | **1** | **1** | **2.23** | **2.11** | **0.018** | **-26** | **-60** | **64** |
| **1** | **0.953** | **19** | **0.71** | **1** | **1** | **2.22** | **2.1** | **0.018** | **20** | **-36** | **50** |
| **1** | **0.953** | **34** | **0.603** | **1** | **1** | **2.2** | **2.08** | **0.019** | **32** | **-42** | **54** |
| **1** | **0.953** | **14** | **0.756** | **1** | **1** | **2.18** | **2.06** | **0.02** | **26** | **-4** | **16** |
| **1** | **0.953** | **87** | **0.389** | **1** | **1** | **2.17** | **2.06** | **0.02** | **-24** | **-96** | **4** |
| **1** | **0.953** | **112** | **0.327** | **1** | **1** | **2.14** | **2.03** | **0.021** | **42** | **-46** | **32** |
|  |  |  |  | 1 | 1 | 2.03 | 1.93 | 0.027 | 40 | -54 | 50 |
|  |  |  |  | 1 | 1 | 2.03 | 1.93 | 0.027 | 38 | -66 | 54 |
| **1** | **0.953** | **20** | **0.701** | **1** | **1** | **2.11** | **2** | **0.023** | **-56** | **8** | **32** |
| **1** | **0.953** | **10** | **0.8** | **1** | **1** | **2.1** | **1.99** | **0.023** | **-12** | **66** | **14** |
| **1** | **0.953** | **24** | **0.67** | **1** | **1** | **2.1** | **1.99** | **0.023** | **52** | **-4** | **4** |
| **1** | **0.953** | **25** | **0.663** | **1** | **1** | **2.08** | **1.97** | **0.024** | **40** | **-68** | **-6** |
| **1** | **0.953** | **10** | **0.8** | **1** | **1** | **2.07** | **1.96** | **0.025** | **-34** | **-18** | **64** |
| **1** | **0.953** | **12** | **0.777** | **1** | **1** | **2.07** | **1.96** | **0.025** | **-20** | **-58** | **30** |
| **1** | **0.953** | **15** | **0.746** | **1** | **1** | **2.03** | **1.94** | **0.026** | **22** | **46** | **30** |
| **1** | **0.953** | **10** | **0.8** | **1** | **1** | **2.02** | **1.92** | **0.027** | **-38** | **-70** | **50** |
| **1** | **0.953** | **13** | **0.766** | **1** | **1** | **2.01** | **1.92** | **0.028** | **-40** | **-2** | **36** |
| **1** | **0.953** | **12** | **0.777** | **1** | **1** | **1.99** | **1.9** | **0.029** | **20** | **-82** | **36** |
| **1** | **0.953** | **14** | **0.756** | **1** | **1** | **1.98** | **1.89** | **0.029** | **40** | **18** | **24** |
| **1** | **0.953** | **14** | **0.756** | **1** | **1** | **1.98** | **1.88** | **0.03** | **-8** | **-10** | **56** |
| **1** | **0.953** | **13** | **0.766** | **1** | **1** | **1.97** | **1.88** | **0.03** | **-44** | **-58** | **6** |
|  |  |  |  | 1 | 1 | 1.89 | 1.81 | 0.035 | -38 | -58 | -2 |
| **1** | **0.953** | **19** | **0.71** | **1** | **1** | **1.94** | **1.86** | **0.032** | **-8** | **28** | **20** |
| **1** | **0.953** | **11** | **0.788** | **1** | **1** | **1.91** | **1.83** | **0.034** | **-48** | **-56** | **48** |
| **1** | **0.953** | **12** | **0.777** | **1** | **1** | **1.9** | **1.82** | **0.035** | **16** | **-76** | **-2** |
| **1** | **0.953** | **12** | **0.777** | **1** | **1** | **1.84** | **1.76** | **0.039** | **-34** | **-42** | **54** |
| **1** | **0.953** | **19** | **0.71** | **1** | **1** | **1.83** | **1.76** | **0.039** | **14** | **-70** | **46** |


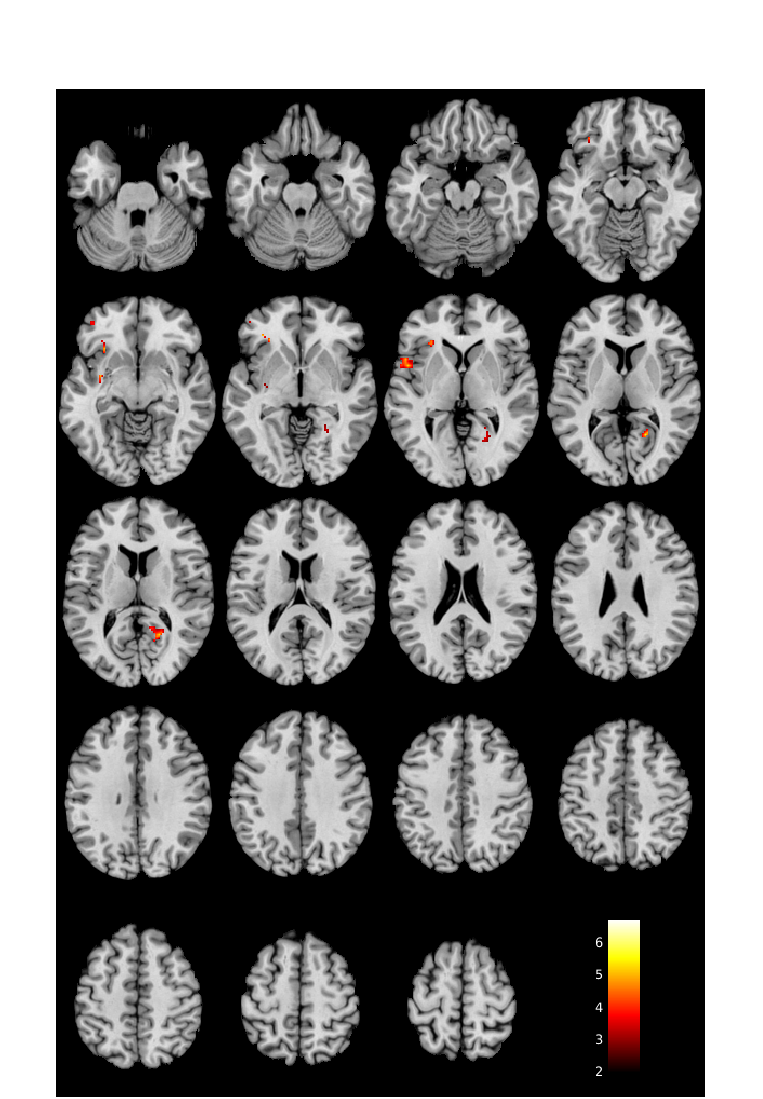


L

R

**Figure S1. Increased activation in NP subjects compared to controls during the pain focus state in contrast to the non-pain focus state using SnPM analysis.** T-statistic mapping of increased bold activation (p<0.05) in NP subjects during the pain focus state compared to the pain minus non-pain contrast in control subjects. A cluster defining threshold of p<0.01 with greater than 300 voxels per cluster was used for display.

**Table S3. Cluster and peak level statistics and MNI coordinates for comparison of increased activation during pain focus state between NP subjects minus controls using SnPM analysis.**

| **Cluster-Level** | | | **Peak-Level** | | | | **mm** | **mm** | **mm** |
| --- | --- | --- | --- | --- | --- | --- | --- | --- | --- |
| **p (FWE)** | **p (uncorr)** | **k** | **p (FWE)** | **p (FDR)** | **T** | **p (uncorr)** |  |  |  |
| **0.1152** | **0.0060** | **339** | **0.3701** | **0.3548** | **6.72** | **0.0010** | **-34** | **-6** | **-4** |
|  |  |  | 0.4648 | 0.3548 | 6.24 | 0.0020 | -50 | 2 | 6 |
|  |  |  | 0.8174 | 0.3548 | 4.75 | 0.0020 | -48 | -4 | 14 |
| **0.1191** | **0.0066** | **316** | **0.3779** | **0.3548** | **6.68** | **0.0010** | **-30** | **18** | **-4** |
|  |  |  | 0.6465 | 0.3548 | 5.50 | 0.0020 | -36 | 30 | -2 |
|  |  |  | 0.7500 | 0.3548 | 5.05 | 0.0020 | -28 | 22 | 4 |
| **0.1133** | **0.0057** | **354** | **0.5283** | **0.3548** | **5.95** | **0.0010** | **20** | **-62** | **12** |
|  |  |  | 0.8750 | 0.3548 | 4.44 | 0.0010 | 16 | -72 | 18 |
|  |  |  | 0.9346 | 0.3548 | 4.02 | 0.0010 | 12 | -54 | 14 |

**Table S4. Cluster and peak level statistics and MNI coordinates for regions of increased activation during pain focus state in NP subjects.**

| **Cluster-Level** | | | | **Peak-Level** | | | | | **mm** | **mm** | **mm** |
| --- | --- | --- | --- | --- | --- | --- | --- | --- | --- | --- | --- |
| **p**  **(FWE)** | **q**  **(FDR)** | **k**  **(E)** | **p**  **(uncorr)** | **p**  **(FWE)** | **q**  **(FDR)** | **T** | **(Z_≡_)** | **p**  **(uncorr)** |  |  |  |
| **0** | **0** | **35085** | **0** | **0.961** | **0.998** | **5.32** | **3.81** | **0** | **28** | **-2** | **14** |
|  |  |  |  | 0.98 | 0.998 | 5.16 | 3.74 | 0 | -48 | 2 | 8 |
|  |  |  |  | 0.98 | 0.998 | 5.16 | 3.74 | 0 | -10 | 44 | -10 |
| **1** | **0.953** | **109** | **0.333** | **1** | **0.998** | **4.02** | **3.18** | **0.001** | **-12** | **-24** | **4** |
|  |  |  |  | 1 | 0.998 | 3.73 | 3.02 | 0.001 | -22 | -26 | 2 |
|  |  |  |  | 1 | 0.998 | 1.88 | 1.74 | 0.041 | -30 | -28 | 0 |
| **1** | **0.953** | **64** | **0.462** | **1** | **0.998** | **3.61** | **2.95** | **0.002** | **-14** | **14** | **8** |
| **1** | **0.953** | **107** | **0.337** | **1** | **0.998** | **3.43** | **2.84** | **0.002** | **36** | **-62** | **-54** |
|  |  |  |  | 1 | 0.998 | 2.3 | 2.07 | 0.019 | 26 | -74 | -52 |
| **1** | **0.953** | **54** | **0.502** | **1** | **0.998** | **3.41** | **2.83** | **0.002** | **-18** | **-28** | **74** |
|  |  |  |  | 1 | 0.998 | 1.87 | 1.74 | 0.041 | -16 | -36 | 66 |
| **1** | **0.953** | **93** | **0.372** | **1** | **0.998** | **3.38** | **2.81** | **0.002** | **12** | **-78** | **-42** |
| **1** | **0.953** | **34** | **0.603** | **1** | **0.998** | **3.22** | **2.71** | **0.003** | **-10** | **20** | **24** |
| **1** | **0.953** | **12** | **0.777** | **1** | **0.998** | **3.16** | **2.67** | **0.004** | **8** | **-48** | **-52** |
| **1** | **0.953** | **48** | **0.529** | **1** | **0.998** | **3.12** | **2.65** | **0.004** | **-12** | **-2** | **40** |
| **1** | **0.953** | **164** | **0.236** | **1** | **0.998** | **3** | **2.57** | **0.005** | **12** | **18** | **32** |
|  |  |  |  | 1 | 0.998 | 2.58 | 2.28 | 0.011 | 18 | 28 | 38 |
|  |  |  |  | 1 | 0.998 | 2.48 | 2.2 | 0.014 | 10 | 16 | 24 |
| **1** | **0.953** | **57** | **0.49** | **1** | **0.998** | **2.98** | **2.55** | **0.005** | **-14** | **54** | **16** |
|  |  |  |  | 1 | 0.998 | 2.47 | 2.2 | 0.014 | -14 | 64 | 14 |
| **1** | **0.953** | **106** | **0.34** | **1** | **0.998** | **2.97** | **2.55** | **0.005** | **38** | **-18** | **40** |
|  |  |  |  | 1 | 0.998 | 2.65 | 2.33 | 0.01 | 36 | -22 | 30 |
| **1** | **0.953** | **10** | **0.8** | **1** | **0.998** | **2.89** | **2.5** | **0.006** | **24** | **-52** | **-48** |
| **1** | **0.953** | **33** | **0.609** | **1** | **0.998** | **2.86** | **2.47** | **0.007** | **46** | **-14** | **54** |
| **1** | **0.953** | **142** | **0.269** | **1** | **0.998** | **2.85** | **2.47** | **0.007** | **-30** | **-72** | **-50** |
|  |  |  |  | 1 | 0.998 | 2.8 | 2.44 | 0.007 | -16 | -74 | -50 |
|  |  |  |  | 1 | 0.998 | 2.57 | 2.27 | 0.012 | -20 | -68 | -42 |
| **1** | **0.953** | **21** | **0.693** | **1** | **0.998** | **2.81** | **2.44** | **0.007** | **18** | **16** | **46** |
| **1** | **0.953** | **42** | **0.559** | **1** | **0.998** | **2.79** | **2.43** | **0.008** | **-44** | **-56** | **-30** |
| **1** | **0.953** | **29** | **0.634** | **1** | **0.998** | **2.75** | **2.4** | **0.008** | **52** | **-10** | **-26** |
| **1** | **0.953** | **20** | **0.701** | **1** | **0.998** | **2.72** | **2.38** | **0.009** | **-16** | **2** | **12** |
| **1** | **0.953** | **17** | **0.727** | **1** | **0.998** | **2.7** | **2.37** | **0.009** | **-22** | **-10** | **30** |
| **1** | **0.953** | **19** | **0.71** | **1** | **0.998** | **2.7** | **2.36** | **0.009** | **2** | **-54** | **-32** |
| **1** | **0.953** | **11** | **0.788** | **1** | **0.998** | **2.69** | **2.36** | **0.009** | **-34** | **-14** | **-18** |
| **1** | **0.953** | **169** | **0.229** | **1** | **0.998** | **2.68** | **2.35** | **0.009** | **0** | **50** | **14** |
|  |  |  |  | 1 | 0.998 | 2.67 | 2.34 | 0.01 | -14 | 40 | 20 |
|  |  |  |  | 1 | 0.998 | 2.5 | 2.22 | 0.013 | 2 | 42 | 18 |
| **1** | **0.953** | **34** | **0.603** | **1** | **0.998** | **2.64** | **2.32** | **0.01** | **18** | **-30** | **54** |
|  |  |  |  | 1 | 0.998 | 2.06 | 1.89 | 0.03 | 20 | -38 | 56 |
| **1** | **0.953** | **25** | **0.662** | **1** | **0.998** | **2.62** | **2.31** | **0.011** | **16** | **10** | **20** |
| **1** | **0.953** | **52** | **0.511** | **1** | **0.998** | **2.62** | **2.31** | **0.011** | **22** | **-62** | **-28** |
|  |  |  |  | 1 | 0.998 | 2.2 | 1.99 | 0.023 | 22 | -66 | -40 |
| **1** | **0.953** | **22** | **0.685** | **1** | **0.998** | **2.45** | **2.18** | **0.015** | **6** | **-62** | **-40** |
| **1** | **0.953** | **16** | **0.736** | **1** | **0.998** | **2.44** | **2.18** | **0.015** | **-4** | **-58** | **-50** |
|  |  |  |  | 1 | 0.998 | 1.78 | 1.67 | 0.048 | 4 | -56 | -46 |
| **1** | **0.953** | **73** | **0.431** | **1** | **0.998** | **2.44** | **2.18** | **0.015** | **12** | **-30** | **28** |
|  |  |  |  | 1 | 0.998 | 2.33 | 2.1 | 0.018 | 0 | -32 | 26 |
| **1** | **0.953** | **20** | **0.701** | **1** | **0.998** | **2.41** | **2.15** | **0.016** | **-50** | **-20** | **-12** |
| **1** | **0.953** | **15** | **0.746** | **1** | **0.998** | **2.39** | **2.14** | **0.016** | **-26** | **30** | **52** |
|  |  |  |  | 1 | 0.998 | 2.2 | 2 | 0.023 | -22 | 36 | 48 |
| **1** | **0.953** | **14** | **0.756** | **1** | **0.998** | **2.32** | **2.09** | **0.018** | **32** | **40** | **42** |
| **1** | **0.953** | **24** | **0.67** | **1** | **0.998** | **2.31** | **2.08** | **0.019** | **30** | **-40** | **50** |
| **1** | **0.953** | **33** | **0.609** | **1** | **0.998** | **2.29** | **2.07** | **0.019** | **2** | **-70** | **-28** |
| **1** | **0.953** | **12** | **0.777** | **1** | **0.998** | **2.27** | **2.05** | **0.02** | **-24** | **-34** | **60** |
| **1** | **0.953** | **21** | **0.693** | **1** | **0.998** | **2.26** | **2.04** | **0.021** | **-44** | **20** | **44** |
|  |  |  |  | 1 | 0.998 | 1.8 | 1.68 | 0.046 | -48 | 24 | 38 |
| **1** | **0.953** | **13** | **0.766** | **1** | **0.998** | **2.26** | **2.04** | **0.021** | **66** | **-20** | **32** |
| **1** | **0.953** | **44** | **0.548** | **1** | **0.998** | **2.23** | **2.02** | **0.022** | **-14** | **-52** | **-18** |
|  |  |  |  | 1 | 0.998 | 2.13 | 1.94 | 0.026 | -6 | -46 | -14 |
| **1** | **0.953** | **15** | **0.746** | **1** | **0.998** | **2.2** | **1.99** | **0.023** | **-54** | **10** | **34** |
| **1** | **0.953** | **20** | **0.701** | **1** | **0.998** | **2.16** | **1.96** | **0.025** | **-22** | **-8** | **20** |
| **1** | **0.953** | **12** | **0.777** | **1** | **0.998** | **2.14** | **1.95** | **0.026** | **-4** | **28** | **4** |
| **1** | **0.953** | **16** | **0.736** | **1** | **0.998** | **2.13** | **1.94** | **0.026** | **0** | **4** | **26** |
| **1** | **0.953** | **12** | **0.777** | **1** | **0.998** | **2.1** | **1.92** | **0.028** | **2** | **24** | **20** |
| **1** | **0.953** | **12** | **0.777** | **1** | **0.998** | **2.09** | **1.91** | **0.028** | **-16** | **-14** | **46** |
| **1** | **0.953** | **10** | **0.8** | **1** | **0.998** | **2.05** | **1.88** | **0.03** | **32** | **34** | **30** |
| **1** | **0.953** | **22** | **0.685** | **1** | **0.998** | **2.01** | **1.85** | **0.032** | **24** | **52** | **26** |
|  |  |  |  | 1 | 0.998 | 1.87 | 1.74 | 0.041 | 32 | 50 | 32 |
| **1** | **0.953** | **30** | **0.628** | **1** | **0.998** | **1.99** | **1.84** | **0.033** | **8** | **-12** | **58** |
| **1** | **0.953** | **10** | **0.8** | **1** | **0.998** | **1.89** | **1.76** | **0.04** | **60** | **-32** | **38** |
|  |  |  |  | 1 | 0.998 | 1.87 | 1.74 | 0.041 | 56 | -30 | 46 |
